# Fast-Acting Caffeine Strategy: A Systematic Review and Meta-Analysis of the Ergogenic Effects of Caffeine Chewing Gum on Physical Performance

**DOI:** 10.1186/s40798-026-01104-y

**Published:** 2026-09-09

**Authors:** Hossein Miraftabi, Erfan Berjisian, Craig Pickering, Alvaro Lopez-Samanes, Olivier Girard

**Affiliations:** 1 Department of Exercise Physiology, Faculty of Sport Sciences and Health, University of Tehran, Tehran, Iran; 2 Perth, WA Australia; 3 Australian Athletics, Melbourne, Australia; 4 GICAF Research Group, Department of Education Research Methods and Evaluation, Faculty of Human and Social Sciences, Universidad Pontificia Comillas, 28049 Madrid, Spain; 5 School of Human Sciences, Exercise and Sport Science, University of Western Australia, Perth, WA 6009 Australia

**Keywords:** Ergogenic aids, Nutrition, Caffeine, Caffeine chewing gum, Exercise performance

## Abstract

**Background:**

Caffeine gum is a fast-acting delivery method that rapidly elevates circulating caffeine levels and may improve physical performance. Given the recent growth in research, an updated systematic review and meta-analysis is warranted.

**Objectives:**

To evaluate the acute effects of caffeine gum on physical performance compared with placebo, and investigate potential moderators including (1) training status, (2) exercise type, (3) chewing duration, (4) caffeine habituation (i.e., habitual intake), (5) caffeine dosage, and (6) timing of administration relative to exercise.

**Methods:**

The review followed the Cochrane Handbook for Systematic Reviews of Interventions and PRISMA guidelines. PubMed, Web of Science, Scopus and SPORTDiscus were searched up to March 2026. Three-level meta-analyses were synthesized for outcomes. Sensitivity analyses addressed assumptions within-subject correlations, outliers, and influential cases. Risk of bias was assessed using standardized tools.

**Results:**

Thirty-one studies were assessed for full-text eligibility, of which 25 (n = 390 participants) met the inclusion criteria and were included in the meta-analysis. Caffeine gum produced a trivial but significant improvement in physical performance (SMD = 0.195, 95% CI [0.115, 0.275], p = 0.001; GRADE: low) with very low heterogeneity (I^2^ = 4%). Three-level variance decomposition indicated that most variability was between studies (64.6%), with no within-study variance. Aerobic endurance showed a small significant effect (p = 0.002), whereas trivial but significant effects were observed for anaerobic performance (p = 0.005) and vertical jump outcomes (p = 0.008). Benefits were evident with a 10-min chewing duration (p = 0.001) for trained individuals (p = 0.018), and among individuals with low (p = 0.003) or moderate (p = 0.013) habitual caffeine intake, but not significant for recreationally active participants (p = 0.676), muscular strength/endurance outcomes (p = 0.372), 5-min chewing duration (p = 0.091), or high habitual caffeine users (p = 0.290). Meta-regression showed a significant dose–response relationship (p = 0.031), whereas the timing of caffeine administration relative to exercise had no effect (p = 0.683). Sensitivity analyses confirmed the robustness of findings across correlation assumptions and leave‑one‑out procedures. Most studies were judged as having some concerns or a high risk of bias. No evidence of publication bias was detected where assessable, although some subgroup analyses could not be formally evaluated because of the limited number of studies. The certainty of the evidence ranged from low to very low.

**Conclusion:**

Caffeine gum produced statistically significant but trivial improvements in physical performance, suggesting limited practical significance. However, even these small improvements may provide a competitive advantage in certain sporting situations.

*Registration* The protocol was prospectively registered in the PROSPERO database (CRD420251086458).

**Supplementary Information:**

The online version contains supplementary material available at https://doi.org/10.1186/s40798-026-01104-y.

## Highlights

Caffeine chewing gum produces a trivial but statistically significant improvement in physical performance, including aerobic endurance, anaerobic, and vertical jump performance. Training status and caffeine habituation influence responses, with greater effects in trained individuals and low–moderate caffeine users, but not in highly habituated or recreational participants. Ergogenic effects are evident with typical doses of ~2–4 mg/kg⁻¹ body mass and chewing durations of 10 min. Due to its rapid buccal absorption and fast onset, caffeine gum is a practical ergogenic option in time-sensitive or competition settings.

**Supplementary Information:**

The online version contains supplementary material available at https://doi.org/10.1186/s40798-026-01104-y.

## Introduction

Caffeine (1, 3, 7-trimethylxanthine) is one of the most widely used ergogenic aids across sports and competitive levels. Since early work over a century ago first demonstrated its performance-enhancing effects on muscular work [1], extensive research has consistently confirmed caffeine’s ergogenic benefits across diverse exercise modalities. Caffeine intake can improve muscular strength and power [2], endurance capacity [3], and performance in intermittent and team sports [4]. These effects are primarily mediated through antagonism of adenosine receptors (A_1_ and A_2_A) in the central nervous system, which increases alertness, promotes arousal, and reduces fatigue perception [5]. Caffeine also stimulates catecholamine release (e.g., adrenaline and noradrenaline) [6], activates dopamine D2 receptors to enhance motivation and reward pathways [7], and lowers pain and effort perceptions during exercise [5], and may increase fat oxidation with consequent glycogen sparing during prolonged aerobic exercise [8]. Additional peripheral mechanisms include enhanced calcium mobilization within muscle fibres during excitation–contraction coupling [9] and improved neuromuscular transmission efficiency [10]. These adaptations may enhance muscle function across strength, power, sprint, and potentially endurance exercise [11, 12]. Together, these central and peripheral actions underpin caffeine’s status as an effective, safe, and widely accessible ergogenic aid. Nevertheless, responses vary considerably between individuals. Factors such as genetic polymorphisms (e.g., CYP1A2, which influences caffeine metabolism, and ADORA2A, which affects caffeine sensitivity), habitual caffeine intake, and expectancy‑related placebo or nocebo effects [13, 14] can all influence metabolism, sensitivity, and perceived performance benefits, thereby contributing to the substantial inter-individual variability reported in the literature. Moreover, recent reviews have highlighted inconsistent findings across studies, suggesting that the magnitude of caffeine’s ergogenic effects may not be uniform across populations or exercise contexts [15, 16].

Traditionally, caffeine is consumed in capsule form [17], with peak absorption occurring 45–60 min after ingestion [3]. In time-constrained scenarios (e.g., warm-ups, early-morning training, or half-time), faster delivery methods are desirable. Caffeine gum has therefore gained attention due to its rapid absorption kinetics [17]. Previous pharmacokinetic studies suggest that caffeine delivered via caffeine gum is partially absorbed through the buccal mucosa, resulting in a faster rise in plasma caffeine concentrations (~ 5–10 min) compared with traditional ingestion methods. However, a considerable proportion is still swallowed and absorbed through the gastrointestinal tract, contributing to its systemic availability and overall pharmacokinetic profile [18, 19]. This rapid availability may be advantageous when the time between ingestion and exercise is limited, although pre‑match ingestion remains the most common strategy to ensure adequate circulating caffeine levels at the start of competition [18]. In addition, some previous studies suggested that caffeine gum may be associated with a lower incidence of commonly reported side effects (e.g., anxiety, headaches, gastrointestinal disorders, and sleep disturbances) compared with traditional forms of ingestion [20].

Numerous studies have investigated the effects of caffeine gum across a range of doses (i.e., 1.35 to 6.4 mg/kg BM) prescribed as relative or absolute doses depending on study design [21, 22], timings of administration (5–120 min before exercise) [23, 24], and exercise protocols (e.g., resistance training, vertical jump, cycling performance, and agility) [23, 25]. Additional factors that have been explored include chewing duration (e.g., 5 vs. 10 min) [26, 27], habitual caffeine intake (low, moderate, and high) [26, 28], and participant characteristics (trained and recreationally active individuals) [19, 29]. Despite the growing body of work on caffeine gum, findings remain inconsistent, likely due to methodological heterogeneity. A comprehensive synthesis is therefore needed to clarify the ergogenic potential of caffeine gum, inform future research, and support evidence-based recommendations for athletes and coaches. An earlier meta-analysis by Barreto et al. [30] demonstrated improvements in both endurance and strength/power performance, supporting caffeine gum as a fast-acting and practical delivery method. Since its publication in 2023, the number of eligible studies on caffeine gum has more than doubled, warranting an updated synthesis.

Several potentially important moderators warrant further investigation. In particular, chewing duration and habitual caffeine intake have not been examined in previous meta-analyses [30], whereas other moderators (e.g., training status, exercise type, caffeine dosage and timing of administration relative to exercise) require re-evaluation in light of the growing evidence base. Furthermore, the unique pharmacokinetic characteristics of caffeine gum, including partial buccal absorption and a more rapid rise in circulating caffeine concentrations than traditional caffeine sources, suggest that these moderators may influence ergogenic responses differently [18]. For example, chewing duration and timing of administration may alter the rate and extent of caffeine absorption, while habitual caffeine intake and dosage may modify performance responses through tolerance-related mechanisms and differences in caffeine bioavailability. Consequently, investigating these moderators may help explain the heterogeneity of findings and identify the conditions under which caffeine gum is most effective [31].

Accordingly, this systematic review and three‑level meta‑analysis aimed to quantify the effects of caffeine gum on exercise performance compared with placebo. Subgroup analyses examined potential moderators, including training status, exercise type, chewing duration, and habitual caffeine intake. Meta‑regression analyses were conducted to further explore the influence of caffeine dose (mg/kg) and timing of administration relative to exercise. By clarifying the magnitude and consistency of these effects across conditions, this work aims to inform the practical use of caffeine gum as a rapid caffeine delivery strategy for performance enhancement.

## Methods

This systematic review and meta-analysis were conducted in accordance with the Cochrane Handbook for Systematic Reviews of Interventions [32] and followed the PRISMA (Preferred Reporting Items for Systematic Reviews and Meta-Analyses) guidelines to ensure transparency and methodological rigour [33]. The protocol was prospectively registered in the PROSPERO database (CRD420251086458). The original registration included both caffeinated chewing gum and caffeine mouth rinsing; however, prior to conducting the review, the scope was refined to include only studies investigating caffeinated chewing gum.

### Eligibility Criteria

This review followed the PICO framework: (1) Participants: healthy adults; (2) Intervention: caffeine gum; (3) Comparison: control condition such as water or non-caloric placebo gum; (4) Outcomes: physical performance. Studies were included if they met all of the following criteria: (1) randomized controlled trials (RCTs) published in peer-reviewed journals; (2) investigated the effects of caffeine gum on physical performance outcomes; (3) involved healthy human participants; (4) included a non-caffeine control condition such as water or non-caloric placebo gum; and (5) reported original experimental data in English. However, for quantitative synthesis, performance outcomes were included only when they could be appropriately assigned to a pre-specified outcome categories and pooled using a comparable standardized effect measure. Outcomes that represented distinct performance constructs or could not be meaningfully harmonized with these categories were excluded from the quantitative synthesis. Exclusion criteria were: (1) interventions involving ingestion or mouth rinsing of caffeine; (2) co-interventions with other active ingredients (e.g. carbohydrate, menthol); (3) studies without exercise performance-related outcomes; (4) reviews, abstracts, or non-original reports; or (5) insufficient methodological information.

### Data Sources and Search

**Fig. 1:**
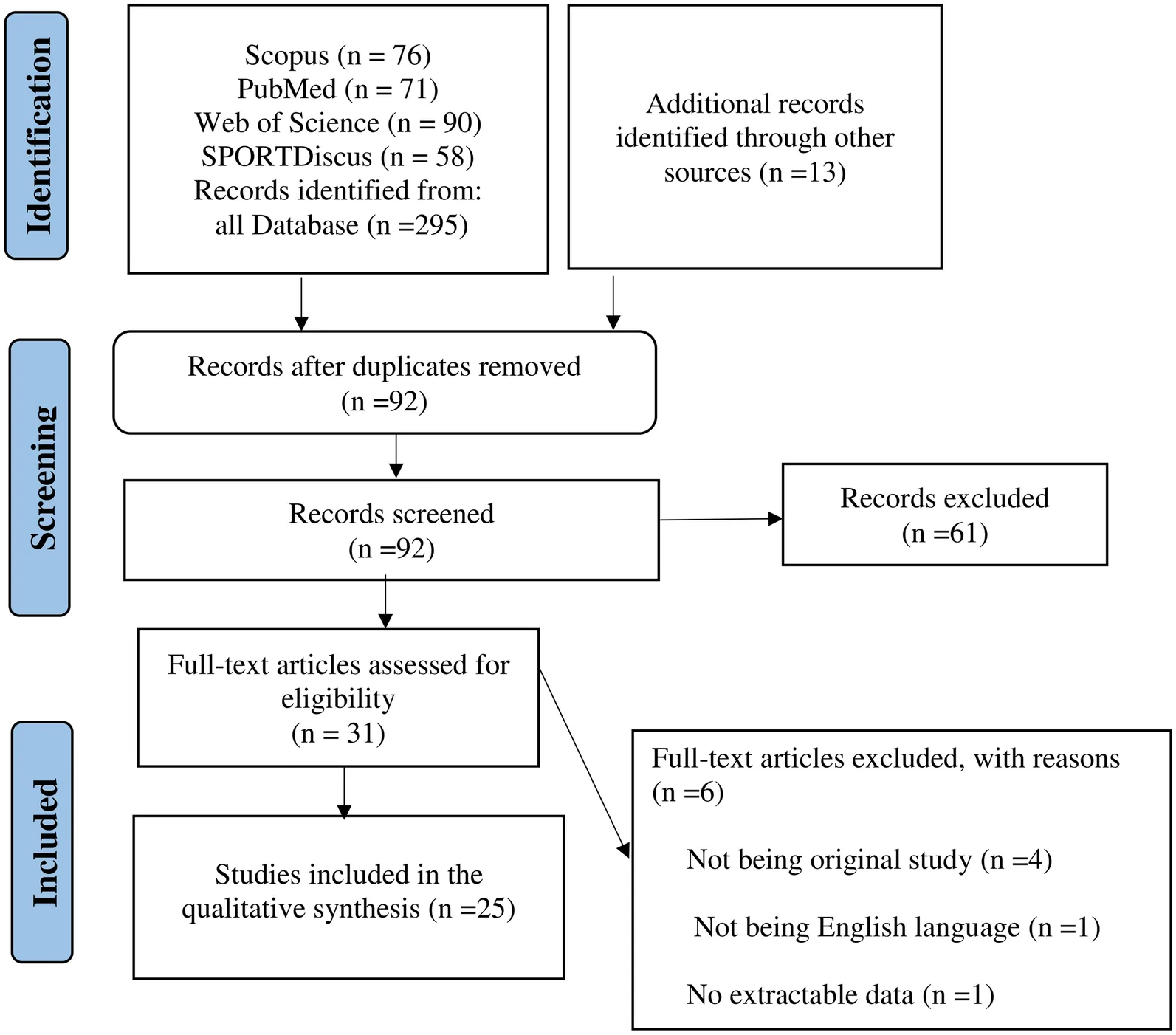
PRISMA 2020 flow diagram of the study selection process

All databases were searched from the start of their indexing period to September 2025, with a final updated search conducted in March 2026. The search was performed across four electronic databases: PubMed, Web of Science, Scopus, and SPORTDiscus using Boolean operators (AND/OR) and free-text keywords. In PubMed, a phrase-based free-text search was used. An example of the search syntax was as follows: ("caffeinated chewing gum" OR "caffeine gum" OR "caffeine chewing gum" OR "caffeinated gum" OR "caffeine-containing chewing gum") AND ("exercise performance" OR exercise OR cycling OR running OR sprint OR "time trial" OR "time to exhaustion" OR endurance OR resistance OR strength OR power). The keyword structure was appropriately adapted for each database. The complete database-specific search strategies, including search strings, search fields, filters, limits, and search dates, are provided in Supplementary Table 3. All retrieved records were imported into EndNote 21 (Clarivate, Philadelphia, PA, USA) for reference management, and duplicates were removed using EndNote’s built-in “Remove Duplicates” function. Following deduplication and title and abstract screening, full-text articles were independently assessed by two authors ([H.M.] and [E.B.]). During full-text screening, the reference lists of eligible studies were also examined to identify any additional relevant articles. The lists of included and excluded studies were then compared for accuracy, and any disagreements were resolved through consultation with another author ([A.S.]). Only peer-reviewed original articles published in English were included; conference abstracts, review articles, and non-peer-reviewed sources were excluded. Grey literature (e.g., conference proceedings, institutional reports), trial registries, preprint servers, and thesis repositories were not systematically searched. The search strategy and study selection process are summarized in the PRISMA flow diagram (Fig. 1).

### Study Selection and Data Collection

For each included study, data were extracted on: (1) title, year, and publication type; (2) study design and participant characteristics, including sample size, sex, age, training status, caffeine habituation, chewing duration, caffeine dose, and timing of administration relative to exercise; and (3) intervention details, including caffeine gum, exercise type, and performance outcomes (i.e., aerobic endurance, anaerobic performance, jump performance, and muscular strength/endurance). All studies reported data as mean ± standard deviation (SD). For one study [21], the corresponding author was contacted to obtain missing data; however, no response was received, and the required information could not be obtained. Consequently, this study was excluded. Although excluding studies because of unavailable data may introduce a risk of bias, only one eligible study was excluded for this reason. Therefore, its impact on the overall pooled estimate is likely to be minimal.

### Quality Assessment

The quality of the included studies was assessed using a modified version of the Quality Criteria Checklist: primary Research [34], following previously published approaches [35]. The quality assessment was independently conducted by two reviewers (H.M. and E.B.) to ensure accuracy and consistency. Any disagreements were resolved through discussion until a consensus was reached.

### Risk of Bias Assessment

A risk of bias assessment was conducted for all studies included in the meta-analysis using the revised Cochrane Risk of Bias tool for randomized trials (RoB 2) [36]. The assessment covered five domains: (1) bias arising from the randomization process; (2) bias due to deviations from intended interventions; (3) bias due to missing outcome data; (4) bias in measurement of the outcome; and (5) bias in selection of the reported result. Each domain was judged as having a low risk of bias, some concerns, or a high risk of bias according to the RoB 2 framework. The assessment was independently carried out by two reviewers (H.M. and E.B.) to ensure accuracy. Any disagreements were discussed and resolved by consensus.

### Statistical Analysis

#### Effect Size Calculation and Data Synthesis

Data analysis and data extraction was performed by H.M. The extracted data and calculated effect sizes were verified by E.B. to ensure accuracy and minimize potential errors. For each included study, mean values, standard deviations (SD), and sample sizes (n) were extracted for meta-analysis (Supplementary Tables 5 and 6). Standardized effect sizes (Hedges’ g) were calculated to account for small-sample bias. For time-based outcomes, where lower values indicate better performance, the direction of the effect size was reversed so that positive SMD values consistently represented performance improvements. When data were available only in graphical form, values were digitized using WebPlotDigitizer (Version 4.3), and corresponding mean and SD values were extracted from the figures [25, 29, 37–39]. All effect size calculations were conducted in accordance with the Cochrane Handbook for Systematic Reviews of Interventions (Version 6.5, 2024) [40]. As all included studies used within-subject crossover designs, the paired nature of the data was accounted for in the effect size calculations [41]. The extracted outcome data were transformed into standardized effect sizes (Hedges’ g) and corresponding sampling variances using the standardized mean change approach (SMCC) implemented in the *metafor* package. Effect-size calculations were based on: (1) mean values for the caffeine gum and placebo conditions, (2) standard deviations for both conditions, (3) sample size (n), and (4) the within-subject correlation between repeated measurements. Hedges’ g was calculated to correct for small-sample bias, while sampling variances were estimated using standard formulas for paired crossover designs that account for the within-subject correlation between conditions. Because none of the included studies reported this correlation, a value of r = 0.50 was assumed for the primary analysis [42]. Effect sizes (g) were also interpreted according to conventional thresholds: trivial (< 0.2), small (0.2–0.5), moderate (0.5–0.8), and large (> 0.8) [43].

#### Three-Level Meta-Analysis and Heterogeneity

To account for multiple outcomes nested within studies, a three-level meta-analysis was performed using the metafor package in R version 4.5.2 for Windows (R Core Team, 2024), with restricted maximum likelihood estimation (REML) [44–46]. The model included random effects at both the study and effect-size levels (random = ~1|study_id/effect_id), accounting for the dependency among multiple effect sizes from the same study. Variance was partitioned into sampling error (level 1), within-study variance (level 2), and between- study variance (level 3) [47]. Accordingly, dependence among multiple outcomes derived from the same sample was addressed through the multilevel modelling framework rather than by averaging outcomes or selecting a single effect size from each study. Heterogeneity was assessed using I^2^ statistics, categorized as low (0–25%), moderate (25–50%), substantial (50–75%), or considerable (> 75%) [42–45, 48]. R code was used to perform the present analyses reported in Supplementary File 4.

#### Moderators and Subgroup Analysis

Moderator analyses were conducted to explore potential sources of variability in effect sizes and to identify conditions under which caffeine gum may influence physical performance. These analyses were performed using weighted random-effects meta-regression models, and moderators were selected based on theoretical relevance and data availability. The examined moderators included training status, exercise type, chewing duration, habitual caffeine intake, caffeine dose, and timing of administration relative to exercise.

Subgroup analysis by sex was not conducted because only one study included female participants (k = 1). Participants’ training status was harmonized into two categories: recreationally active (including recreationally active or physically active participants) and trained (including trained or highly trained participants). This harmonization reduced category fragmentation while preserving meaningful differences in training level.

Exercise outcomes were classified into four categories based on the predominant physiological demands of the performance task: jump performance (e.g., countermovement jump and Sargent jump), aerobic endurance (e.g., 5-km running, Yo-Yo Intermittent Recovery Test, cycling time trials, and time-to-exhaustion protocols), anaerobic performance (e.g., sprint tests, agility tests, rowing ergometer tests, and RAST), and muscular strength and endurance (e.g., 1RM tests, repetitions to failure, handgrip strength, and Romanian deadlift tests [49]). A detailed classification of exercise outcomes and the justification for their categorization are also provided in Supplementary Table 2. Each outcome was considered separately and assigned to the category that best reflected its primary performance demand. Sport-specific outcomes were included when they could be reasonably classified within a pre-specified category. Distinct or highly task-specific outcomes, such as rate of force development, time to peak torque, barbell velocity, and technical skills or accuracy measures, were excluded from the quantitative synthesis to maintain conceptual comparability and minimize heterogeneity.

Chewing duration was categorized as short exposure (5 min) or long exposure (10 min) based on the most frequently reported durations across studies. Habitual caffeine intake was also estimated using reference values from the U.S. Department of Agriculture Food Data Central database and categorized as low (0–150 mg/day), moderate (150–300 mg/day), high (> 300 mg/day), or unclear when intake was not reported [50].

Caffeine dose was analyzed to explore potential dose–response relationships and was treated as a continuous moderator because the reported relative doses (mg/kg) showed substantial variability across the included studies. Therefore, rather than categorizing doses into arbitrary groups, the full range of reported values was retained to capture better potential linear associations between caffeine dose and performance outcomes.

Timing of caffeine administration relative to exercise was analyzed as a continuous moderator, given that the included studies reported a wide range of ingestion times before exercise. Treating this variable as continuous enabled the analysis to capture variability in timing protocols across studies and to more closely examine potential relationships between the timing of caffeine gum administration and performance outcomes.

Mixed‑effects multilevel meta‑regression models were fitted using restricted maximum likelihood (REML) estimation with the rma.mv function from the *metafor* package. Effect sizes were nested within studies to account for statistical dependence among multiple outcomes within the same study, and statistical inference for moderators was obtained from model‑based tests within the multilevel framework. Continuous moderators, including caffeine dose (mg/kg) and the timing of administration relative to exercise, were entered as continuous predictors in the meta‑regression models. Categorical moderators (e.g., training status, exercise type, chewing duration and caffeine habituation) were included as factor variables, and models were specified with an intercept to assess differences in effect sizes between categories [51, 52]. Forest plots were generated using the forest function from the *metafor* package in R. Effect sizes were reported as standardized mean differences (SMDs) with their corresponding confidence intervals (CIs). Study labels included the subgroup analysis, and additional study information (n = number of participants and k = number of effect sizes) was presented alongside each effect size estimate.

#### Publication Bias and Sensitivity Analyses

Publication bias was assessed using Egger’s regression test for outcomes with at least ten effect sizes. Several sensitivity analyses were conducted to evaluate the robustness of the findings. First, leave-one-out analyses were performed to examine the influence of each individual study on the pooled estimates for both the overall effect and subgroup analyses. Additional sensitivity analyses were conducted for categorical moderators (e.g., training status, exercise type, chewing duration, and caffeine habituation) as well as for continuous moderators, including caffeine dose (mg/kg) and the timing of administration relative to exercise. Because several included studies used crossover designs, sensitivity analyses were also performed using alternative assumed within-subject correlations (r = 0.20 and r = 0.80) to evaluate the robustness of the meta-analytic estimates.

#### Certainty of Evidence

The certainty of the evidence was assessed using the GRADE (Grading of Recommendations Assessment, Development, and Evaluation) framework. This approach considers several domains, including risk of bias, inconsistency, indirectness, imprecision, and publication bias [53]. Based on these criteria, the certainty of evidence was classified as high, moderate, low, or very low. All GRADE assessments were initially performed by one reviewer and subsequently checked by a second reviewer. Any discrepancies were resolved through discussion until consensus was achieved.

## Results

### Systematic Review Results

#### Search Results

A total of 25 studies were included in the current meta-analysis. These studies provided 47 effect size estimates (k = 47) that examined physical performance. The main findings and methodological characteristics of the included studies are summarized in Table 1.

**Table 1:** Summary of studies included in the meta-analyses for caffeinated chewing gum (n = 25)

Authors	Exercise protocol	Population (n)	Chewing duration (min)	Mean age (y); body weight (kg); caffeine habituation status	Dose (mg/kg)	Time of consumption before exercise (min)	Main outcomes
Li Ding et al. [54] RDB Crossover	1 RM bench press/squat	Male/trained/resistance training (16)	5 min	20.8 ± 1.6; 72.9 ± 6.1; Low	4 (mg/kg)	5 min	Bench press ↑ Squat ↑ (p < 0.05)
Li Ding et al. [38] RDB Crossover	1 RM bench press/squat	Male/trained/resistance training (16)	5 min	21.6 ± 2.0; 79.6 ± 8.8; Low	3 (mg/kg)	5 min	Bench press ↑ Squat ↑ (p < 0.05)
Li Ding et al. [55] RDB Crossover	1 RM bench press/squat	Male/trained/resistance training (16)	5 min	21.6 ± 2.0; 79.6 ± 8.8; Low	4 (mg/kg)	10 min	Bench press ↔ (p > 0.05) Squat ↑ (p < 0.05)
Teimouri-Korani et al. [25] RDB Crossover	Sargent	Male/trained/resistance training (15)	10 min	25 ± 4; 75 ± 11; Low	3–4.5 (mg/kg)	60 min	Sargent ↑ (p < 0.05)
Lynn et.al [29] RDB Crossover	5 km running	Male/recreational/running (14)	5 min	33.7 ± 10.7; NA; Low	2.48–3.79 (mg/kg)	30 min	5 km running ↑ (p < 0.05)
Tsai et al. [27] RDB Crossover	Yo-Yo IR1/CMJ/35 m sprint	Elite/ice hockey (14)	10 min	25.2 ± 5.4; 78.1 ± 13.4; NA	3 (mg/kg)	Immediately before warm-up	Yo-Yo IR1 ↔ 35 m sprint ↔ (p > 0.05) CMJ ↑ (p < 0.05)
Tallis et al. [26] RDB Crossover	CMJ	Male/rugby (27)	5 min	20 ± 2; 96.6 ± 18.2; Moderate	3 (mg/kg)	10 min	CMJ ↔ (p > 0.05)
Yi-Jie Shiu et al. [56] RDB Crossover	400 m sprint	Male/trained/sprinting (19)	10 min	20.9 ± 1.0; 66.5 ± 5.6; NA	3 (mg/kg)	15 min	400 m sprint ↑ (p < 0.05)
Liu et al. [28] RDB Crossover	CMJ/ T-agility test/ RAST	Male/trained/basketball (15)	10 min	20.9 ± 1.0; 77.2 ± 7.5; Low	3 (mg/kg)	15 min	CMJ/ T-agility ↔ (p > 0.05) RAST ↑ (p < 0.05)
Farmani et al. [57] RDB Crossover	Bruce / Sargent	Male/professional/table tennis (18)	10 min	21.86 ± 2.40; 61.81 ± 10.32; Low	3–4.5 (mg/kg)	10 min	Bruce ↑ (p < 0.05) Sargent ↔ (p > 0.05)
Pirmohammadi et al. [58] RDB Crossover	Edgren’s agility test	Female/professional/table tennis (18)	10 min	20.50 ± 3.05; 56.83 ± 6.50; NA	3–4.7 (mg/kg)	10 min	Edgren’s agility test ↑ (p < 0.05)
Yildirim et al. [22] RDB Crossover	15 s CMJ	Male/trained/soccer (14)	5 min	22 ± 2; 74.2 ± 7.1; High	2.68 or 1.34 (mg/kg)	10 min	15 s CMJ ↔ (p > 0.05)
Kaszuba et al. [59] RDB Crossover	CMJ/ T-agility test/10 m sprint	Male, female/trained/volleyball (12)	5 min	23 ± 3; 85.9 ± 11.2; Moderate	3.2 (mg/kg)	15 min	CMJ ↔ T-agility test ↔ 10 m sprint ↔ (p > 0.05)
Filip Stachnik et al. [60] RDB Crossover	Special judo fitness test	Male/elite/judo (9)	5 min	23.7 ± 4.4; 73.5 ± 7.4; Moderate	2.7 or 5.4 (mg/kg)	15 min	Special judo fitness test ↔ (p > 0.05)
Dittrich et al. [61] RDB Crossover	Treadmill run at 15.4 ± 0.7 km/h	Male/well-trained/running (12)	5 min	31.3 ± 6.4; 70.5 ± 6.6; Low	4.26 (mg/kg)	NA	Treadmill run test ↑ (p < 0.05)
Venier et al. [62] RDB Crossover	CMJ/ Rowing ergometer peak power output	Male/trained/resistance training (19)	10 min	24 ± 5; 83 ± 10; Low	3.61 (mg/kg)	Before starting the tests	CMJ↑ Rowing ergometer peak power output ↑ (p < 0.05)
Ranchordas et al. [63] RDB Crossover	CMJ/ Yo-Yo IR2/Illinois agility test/6×30 m sprint	Male/trained/rugby (17)	5 min	20.4 ± 1.2; 85.6 ± 6.3; NA	2.3 (mg/kg)	After the warm-up	CMJ ↑ Yo-Yo IR2 ↑ (p < 0.05) 6×30 m sprint ↔ Illinois agility test ↔ (p > 0.05)
Ranchordas et al. [64] RDB Crossover	20 m sprint/ Yo-Yo IR1/CMJ	Male/trained/soccer (10)	5 min	19 ± 1; 75.5 ± 4.8; NA	2.7 (mg/kg)	5 min	Yo-Yo IR1 ↑ CMJ ↑ (p < 0.05) 20 m Sprint ↔ (p > 0.05)
Russell et al. [39] RDB Crossover	2 Repeated sprint tests: 6 × 40 m sprints	Male/professional/rugby (14)	5 min	18 ± 1; 98.6 ± 10.9; Moderate	4.1 (mg/kg)	15 min	2 Repeated sprint tests: 6 × 40 m sprints ↔ (p > 0.05)
Evans et al. [24] RDB Crossover	10×40 m Maximal shuttle run test	Male/team sport (18)	10 min	21.2 ± 1; 80.4 ± 6.6; Low and Moderate	2.5 (mg/kg)	5 min	10×40 m maximal shuttle run test (Moderate) ↔ (p > 0.05) 10×40 m maximal shuttle run test (Low) ↑ (p < 0.05)
Ryan et al. [23] RDB Crossover	Cycling to volitional exhaustion	Male/recreational/cycling (8)	5 min	25 ± 5; NA; Moderate	2 (mg/kg)	120, 60, 5 min	Cycling to volitional exhaustion ↔ (p > 0.05)
Paton et al. [19] RDB Crossover	30-km cycling time trial	Trained/cycling (20)	5 min	30 ± 10; 69 ± 10; Moderate	3–4 (mg/kg)	NA	First 20-km ↔ (p > 0.05) Final 10-km ↑ (p < 0.05)
Chen et al. [37] RDB Crossover	Romanian deadlift	Recreational/Resistance training (19)	5 min	22.5 ± 3.5; 78.8 ± 13.2; Low	2.5 (mg/kg)	10 min	Romanian deadlift performance on the flywheel machine ↑ (p < 0.05)
Tzeng et al. [65] RDB Crossover	Hand grip strength	Male/trained/wrestler (16)	10 min	21.8 ± 1.0; 68.2 ± 8.7; Low	3 (mg/kg)	15 min	Grip strength ↔ (p > 0.05)
Vargas-Molina et al. [66] RDB Crossover	Total number of repetitions (pull-ups, push-ups, and squats) CMJ	Male/CrossFit^®^ (14)	NA	30.9 ± 5.62; 78 ± 5.75; NA	3 (mg/kg)	NA	Total number of repetitions ↔ (p > 0.05) CMJ ↔ (p < 0.05)

*RDB* Randomized double-blind crossover trial, *CMJ* Countermovement jump, *Yo-Yo IR1* Yo-Yo Intermittent Recovery Test Level 1, *Yo-Yo IR2* Yo-Yo Intermittent Recovery Test Level 2, *RAST* Running-Based Anaerobic Sprint Test, *RM* Repetition maximum, *NA* Not available or not applicable, *min* Minutes, *kg* Kilograms, *mg/kg* Milligrams per kilogram of body mass, *y*: Years, *↑* Significant improvement following caffeine gum ingestion, *↔* No significant difference between caffeine gum and placebo

#### Characterization of Participants

Across all studies, a total of 390 participants were included (359 males, 31 females), with sample sizes ranging from 8 to 27. Most of exercise performance studies recruited only males (n = 22, k = 42), two studies recruited mixed-sex samples (k = 4), and one study recruited exclusively female participants. For training level, 20 studies (k = 40) included trained participants, four studies (k = 6) included recreationally active individuals, and one study did not specify (k = 1).

According to the type of exercise categorized by the primary underlying energy system or neuromuscular mechanism, nine studies investigated aerobic endurance (k = 11), 10 examined anaerobic performance (k = 14), 12 assessed jump (k = 13), and six evaluated muscular strength and endurance (k = 9).

Chewing duration was categorized according to the time of chewing before expectoration, with studies grouped into short exposure (5 min; n =12, k =24) and longer exposure (10 min; n = 10, k = 17) conditions. Three studies did not report the time of chewing gum (k = 6).

Some studies did not report participants’ habitual caffeine intake (n = 6, k = 13). Among those that did, the majority primarily included low‑caffeine consumers (n = 10, k = 20), whereas eight studies involved participants with moderate caffeine intake (n = 8, k = 12). Notably, only one study recruited high-caffeine consumers (n = 1, k = 2). Questionnaire-based methods were the most common approach for assessing habitual caffeine intake (k = 16, n = 9), followed by dietary records (k = 4, n = 2), survey-based assessments (k = 1, n = 1), and website/database estimation tools (k = 1, n = 1). However, the assessment method was not reported in a considerable number of studies (k = 25, n = 12).

Caffeine doses reported as absolute values were converted to relative doses (mg/kg) using the mean body mass of the respective study samples to ensure comparability across trials. Caffeine dose ranged from 1.35 to 6.4 mg/kg across studies. Most effect sizes were derived from doses between 2 and 4 mg/kg, with values around 3 mg/kg being most frequently reported.

Timing of caffeine administration relative to exercise was predominantly clustered at shorter pre‑exercise intervals, with most effect sizes reported for ingestion within 0–10 min before exercise (commonly 5 and 10 min). Fewer observations were available at 15 min, and only isolated cases were reported at longer intervals (30–35 min and 60 min before exercise). For more details, please refer to Table 1.

#### Meta‑Analysis

Analysis of 47 outcomes using a multilevel meta-analytic model indicated a trivial but statistically significant positive effect of caffeine gum on physical performance compared with placebo (k = 47, n = 390, SMD = 0.195, 95% CI [0.115 to 0.275], p = 0.001, GRADE: low). Heterogeneity across studies was very low (I^2^ = 4% [low], Q = 37.53, df = 46, p = 0.809). Variance decomposition from the three‑level meta‑analytic model indicated that 64.6% of the total variability was attributable to differences between studies (Level 3; τ^2^ = 0.002), while no variability was observed at the within‑study level (Level 2; τ^2^ = 0.000; 0%). Nevertheless, moderator analyses were conducted to further explore potential sources of variability across studies.

#### Moderator Analysis

The overall test of moderators indicated that training status significantly moderated the effect of caffeine gum on exercise performance (QM (1) = 5.587, p = 0.018). Caffeine gum produced a trivial, non‑significant effect in recreationally active participants (k = 6, n = 59, SMD = −0.038, 95% CI [− 0.244, 0.167], p = 0.676, I^2^ = 26% [moderate], GRADE: Very low) [23, 24, 29], whereas a small but statistically significant effect was observed in trained participants (k = 40, n = 304, SMD = 0.228, 95% CI [0.146, 0.310], p = 0.018, I^2^ = 0% [low], GRADE: Low) [19, 22, 25–28, 38, 39, 54–66].

Exercise outcome did not significantly moderate the ergogenic effects of caffeine gum (QM (3) = 0.864, p = 0.834). Nevertheless, subgroup analyses showed small but statistically significant improvements in aerobic endurance outcomes (k = 11, n = 122, SMD = 0.258, 95% CI [0.081, 0.436], p = 0.002, I^2^ = 0% [low], GRADE: Very low) [19, 23, 27–29, 57, 61, 63, 64] and trivial but statistically significant improvements in anaerobic performance (k = 14, n = 156, SMD = 0.186, 95% CI [0.042, 0.340], p = 0.005, I^2^ = 0% [low], GRADE: Very low) [24, 27, 28, 39, 58–60, 63, 64, 67] and jump performance (k = 13, n=195, SMD = 0.183, 95% CI [0.037, 0.330], p = 0.008, I^2^ = 0% [low], GRADE: Very low) [26–28, 57, 59, 63, 64, 66, 68]. In contrast, the effect for muscular strength and endurance was trivial but non-significant (k = 9, n = 97, SMD = 0.142, 95% CI [− 0.038, 0.322], p = 0.372, I^2^ = 35% [moderate], GRADE: Very low) [25, 37, 38, 54, 55, 65, 66].

Chewing duration (5 vs. 10 min) was not a significant moderator (QM (1) = 2.154, p = 0.142). However, a small, significant effect was noted following 10 min of chewing (k = 17, n = 190, SMD = 0.262, 95% CI [0.151, 0.384], p = 0.001, I^2^ = 0% [low], GRADE: low) [24–28, 56–58, 62, 65], whereas the effect after 5 min was trivial and non-significant (k = 24, n =110, SMD = 0.142, 95% CI [−0.030, 0.262], p = 0.091, I^2^ = 7% [low], GRADE: Very low) [19, 29, 37–39, 55, 59–61, 63, 64, 69].

**Fig. 2:**
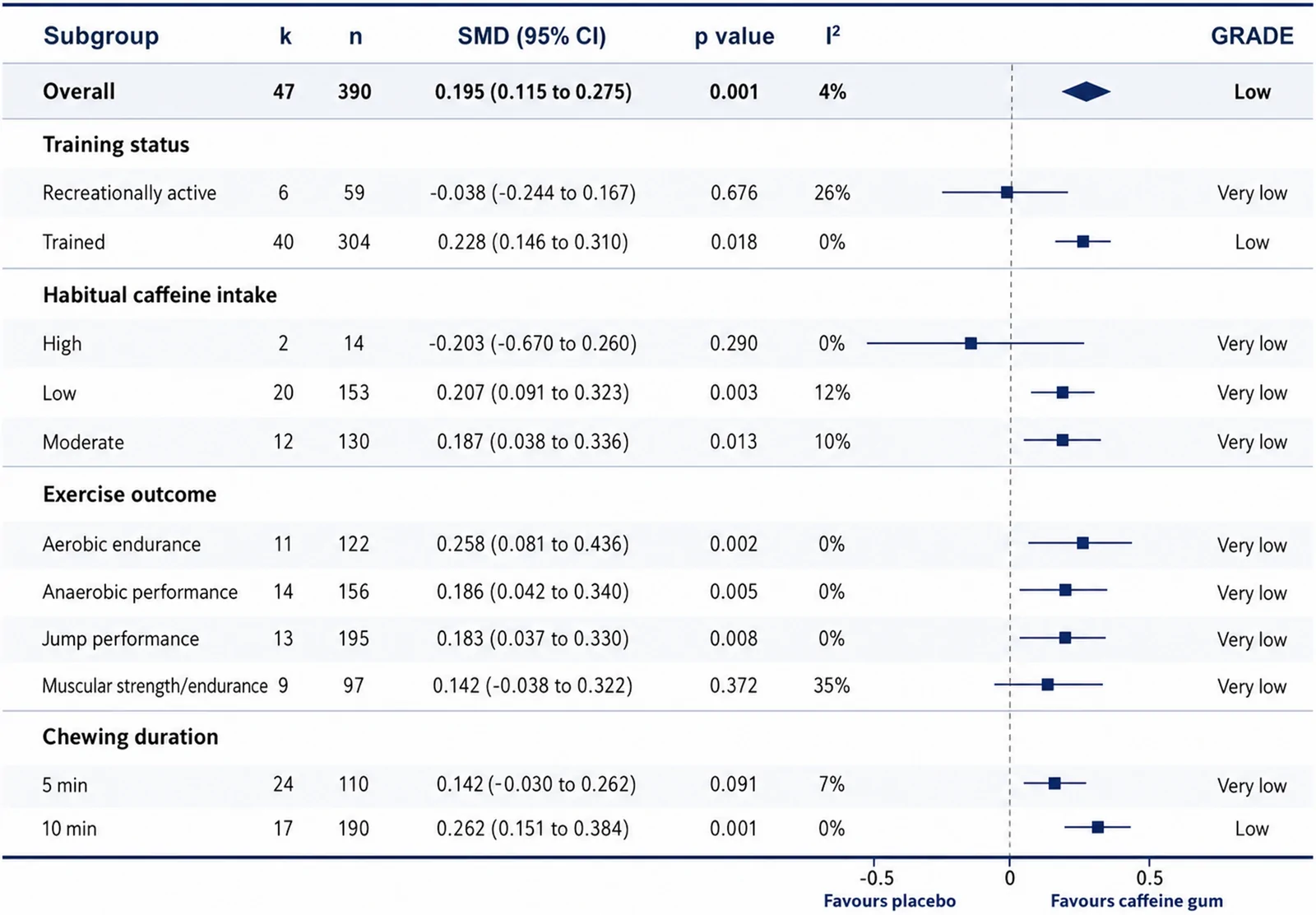
Moderator analysis for physical performance results. k, the total number of effects included in the pooled effect size; SMD, the effect size indicators used in the pooled; 95% CI, 95% confidence interval; P-value, statistically significant P values for pooled results; I^2^, quantitative indicators of heterogeneity; GRADE, grading of recommendations assessment, development, and evaluation, a system for evaluating the quality of evidence and strength of recommendations

Habitual caffeine intake also did not significantly moderate the ergogenic effects of caffeine gum (QM (2) = 3.465, p = 0.176). A small, non-significant reduction in performance was observed among individuals with high habitual caffeine intake (k = 2, n = 14, SMD = −0.203, 95% CI [− 0.670, 0.260], p = 0.290, I^2^ = 0% [low], GRADE: very low) [22]. In contrast, small but statistically significant improvements were observed in participants with moderate habitual caffeine intake (k = 12, n = 130, SMD = 0.187, 95% CI [0.038, 0.336], p = 0.013, I^2^ = 10% [low], GRADE: Very low) [24, 25, 38, 54, 55, 62, 64, 69, 70] and low habitual caffeine intake (k = 20, n = 153, SMD = 0.207, 95% CI [0.091, 0.323], p = 0.003, I^2^ = 12% [low], GRADE: Very low) [19, 24, 26, 39, 57, 59, 61, 71]. A summary of all subgroup analyses is presented in Fig. 2.

A meta‑regression analysis was conducted to examine whether caffeine dose moderated the effect size. The results indicated a significant positive association between caffeine dose and effect size (β = 0.100, p = 0.031, 95% CI [0.002, 0.192]). Residual heterogeneity for this model was not significant (QE (44) = 31.17, p = 0.927). Meta‑regression analysis showed that timing of administration relative to exercise was not a significant moderator of the effect size. The regression coefficient indicated a non‑significant association between timing of administration relative to exercise and performance outcomes (β = −0.002, p = 0.683, 95% CI [− 0.010, 0.006]). Residual heterogeneity was not significant as well (QE = 32.38, p = 0.799).

#### Sensitivity Analysis

##### Sensitivity Analysis for Primary Effect

Sensitivity analyses using different assumed within‑subject correlations (r = 0.2 and r = 0.8) yielded consistent findings for overall effect size. Under these assumptions, the ergogenic effect of caffeine gum remained significant (r = 0.2: SMD = 0.156, p < 0.001; r = 0.8: SMD = 0.282, p < 0.001). A leave‑one‑study‑out sensitivity analysis also was conducted to assess the robustness of the pooled effect. The results indicated that the overall effect size remained stable when each study was removed in turn, with pooled estimates ranging from SMD = 0.182 to SMD = 0.215. All models remained statistically significant (all p < 0.001). A sensitivity analysis excluding Ding et al. [38], owing to potentially overlapping participant characteristics with another study from the same research group, yielded a similar pooled effect estimate (SMD = 0.183, 95% CI [0.103, 0.263]), indicating that the overall findings were robust to potential sample overlap. In addition, heterogeneity remained very low across all iterations, with I^2^ values ranging from 0% to 7.51%.

##### Sensitivity Analysis for Moderator Effect

Sensitivity analyses using different assumed within-subject correlations (r = 0.2 and 0.8) yielded consistent findings for the training status subgroup. A significant ergogenic effect of caffeine gum was observed in trained participants across all correlation assumptions (r = 0.2: SMD = 0.184, p = 0.001; r = 0.8: SMD = 0.344, p < 0.001). In contrast, no significant effects were observed in recreationally active individuals (r = 0.2: SMD = −0.034, p = 0.746; r = 0.8: SMD = −0.083, p = 0.595). Leave-one-out sensitivity analysis showed that results in the trained subgroup were highly robust, and all models remained statistically significant. In contrast, in the recreationally active subgroup the effect size varied slightly although remained non-significant in all models.

Sensitivity analyses using different assumed within‑subject correlations (r = 0.2 and 0.8) showed consistent findings across exercise modes. Significant ergogenic effects of caffeine gum were observed for aerobic endurance (r = 0.2: SMD = 0.212, p = 0.013; r = 0.8: SMD = 0.402, p = 0.001), anaerobic performance (r = 0.2: SMD = 0.153, p = 0.025; r = 0.8: SMD = 0.271, p = 0.008), and jump performance (r = 0.2: SMD = 0.156, p = 0.037; r = 0.8: SMD = 0.293, p = 0.004). In contrast, no significant effect was observed for muscle strength and endurance tasks (r = 0.2: SMD = 0.121, p = 0.158; r = 0.8: SMD = 0.161, p = 0.124). Leave-one-out sensitivity analyses across exercise-type subgroups showed that the pooled effects for aerobic endurance, jump, and anaerobic performance remained stable and statistically significant after removing individual studies, indicating robust results. In contrast, the muscle strength and endurance subgroup showed some variability, with the effect becoming significant after the removal of one study [37], suggesting sensitivity to individual studies.

Across all assumed within-subject correlations (r = 0.2 and 0.8), chewing duration demonstrated consistent effects. At r = 0.2, the 10 min condition showed a significant ergogenic effect (SMD = 0.210, p = 0.001), whereas the 5 min condition was non-significant (SMD = 0.120, p = 0.121). At r = 0.8, effect sizes were larger for both conditions, with significant improvements observed for 10 min (SMD = 0.393, p = 0.001) and no significant effect was revealed for 5 min (SMD = 0.210, p = 0.065). Leave-one-out sensitivity analyses for caffeine gum chewing duration showed different patterns between conditions. For the 10 min condition, the pooled effect size remained stable and statistically significant after removing individual studies, indicating robust results. In contrast, the 5 min condition exhibited greater variability in the leave-one-out analyses. Although the overall effect remained statistically non-significant, the magnitude of the estimate fluctuated when individual study [37] was removed, indicating some sensitivity to study-level influence.

Using alternative assumed correlations (r = 0.2 and r = 0.8) did not materially alter the conclusions of the habituation subgroup analysis. For low habitual caffeine intake, the ergogenic effect remained small but statistically significant across both correlations (SMD range: 0.165 to 0.289; all p < 0.010), indicating high robustness. For the moderate habituation subgroup, effects were likewise stable and consistently significant (SMD range: 0.153 to 0.308). Leave‑one‑out sensitivity analyses for the low and moderate habitual caffeine intake subgroups showed that the effects were highly robust, remaining stable and statistically significant across all iterations.

Meta‑regression analyses examined the association between caffeine gum dose (mg/kg) and performance outcomes across different assumed within‑subject correlations. For r = 0.2, the relationship between dose and effect size showed a non‑significant trend (β = 0.083, p = 0.063), while for r = 0.8, a significant positive dose–response relationship was observed (β = 0.162, p = 0.008), indicating significantly larger ergogenic effects with increasing caffeine dose. A leave-one-out sensitivity analysis was conducted to examine the robustness of the meta-regression for caffeine dose (mg/kg). The results showed the effect remained statistically significant in nearly all iterations, except when study [22] and study [37] were removed, where the association was no longer statistically significant.

Across the sensitivity analyses using r = 0.2 and r = 0.8, meta-regression indicated that the timing of administration relative to exercise was not significantly associated with performance outcomes. The regression coefficients (r = 0.2: β = −0.001, p = 0.712; r = 0.8: β = −0.002, p = 0.702), suggesting that variations in timing of administration relative to exercise did not meaningfully influence the ergogenic effect. A leave-one-out sensitivity analysis was also conducted for the meta-regression examining the timing of administration relative to exercise. The results showed that the regression coefficient remained relatively stable across iterations and the association remained statistically non-significant after the removal of individual studies.

#### Risk of Bias and Quality of Methods

**Fig. 3:**
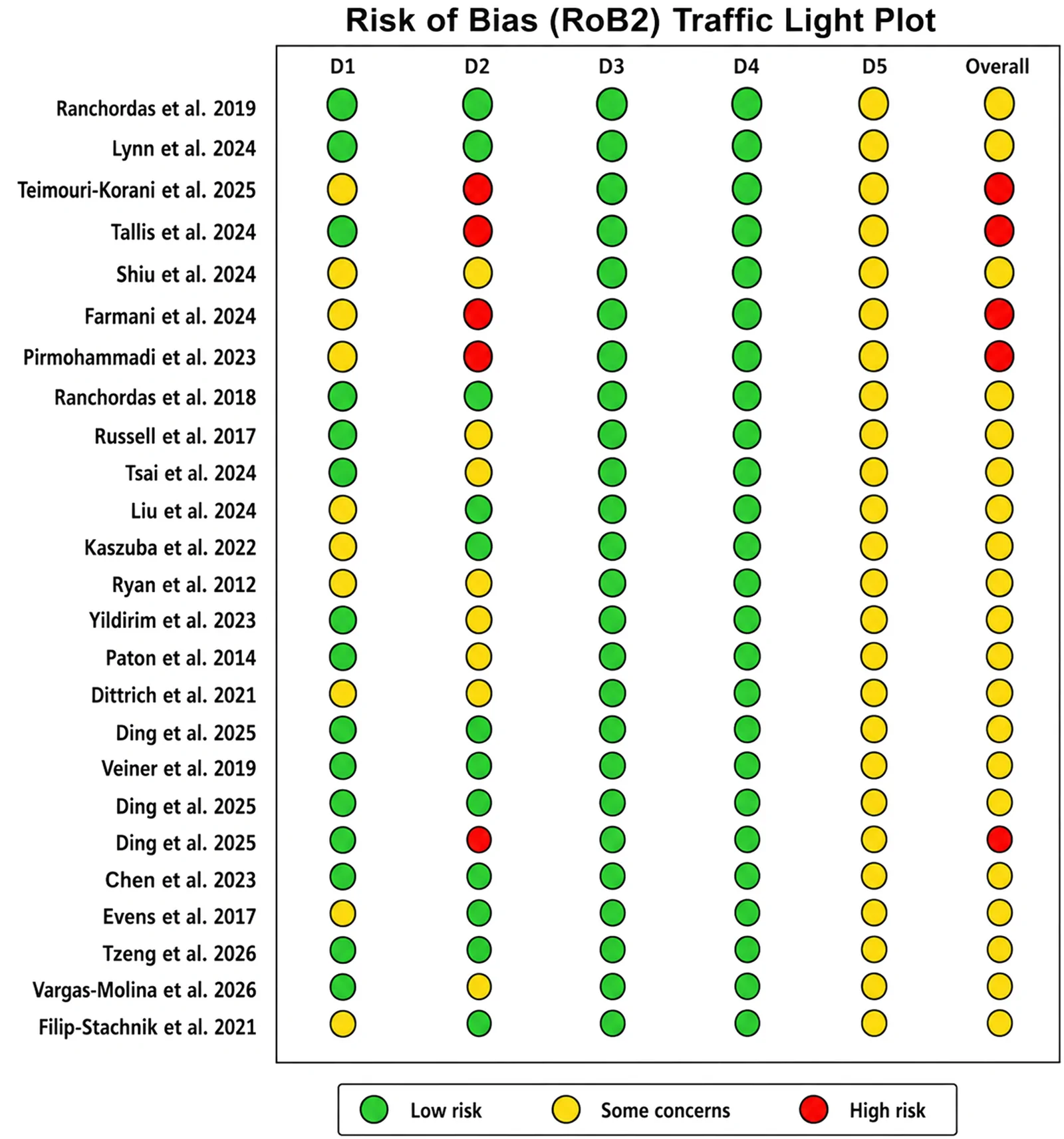
Risk of bias for caffeinated chewing gum

Across the included studies, most domains were judged as low risk of bias, particularly for missing outcome data (D3) and measurement of outcomes (D4). However, Domains 1 (randomization process) and 5 (selection of the reported result) showed the highest proportion of some concerns, mainly due to insufficient reporting of randomization methods and lack of pre‑specified analysis plans. A high risk of bias was observed in a few studies in Domain 2 (deviations from intended interventions), primarily related to unclear blinding procedures (Fig. 3).

**Fig. 4:**
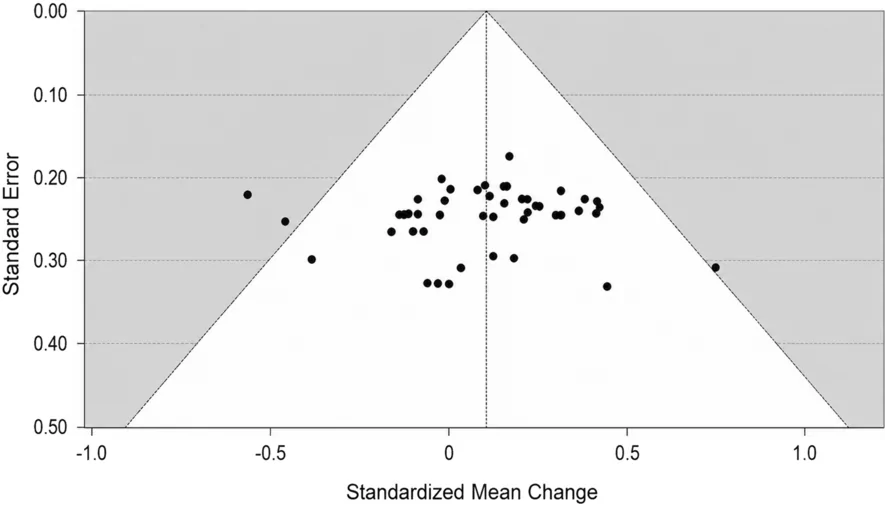
Funnel plot for assessment of publication bias across the included studies

The mixed-effects meta-regression model assessing funnel plot asymmetry showed no statistically significant evidence of publication bias (Fig. 4). Egger’s regression test yielded a non-significant result (z = −0.19, p = 0.848), indicating that the relationship between effect size and its standard error was not systematic. The limit estimate as the standard error approached zero was b = 0.251 (95% CI: −0.33, 0.84). However, these findings should be interpreted with caution because Egger’s test has limited statistical power when the number of independent studies is small, and its interpretation may be further complicated by the inclusion of dependent effect sizes. Therefore, the absence of statistically significant funnel plot asymmetry should not be interpreted as definitive evidence that publication bias is absent. Egger’s regression tests were conducted to assess potential publication bias across all examined subgroups. Egger’s tests across subgroups based on training status, exercise type, caffeine habituation level, and chewing duration also showed no significant funnel plot asymmetry (all p > 0.05), suggesting no evidence of publication bias. However, publication bias could not be formally assessed for several subgroup analyses because of the limited number of available studies.

Additionally, Egger’s regression tests conducted for meta regression models including caffeine gum dose and timing of administration relative to exercise as moderators showed no significant funnel plot asymmetry (caffeine dose: z = −0.67, p = 0.502; timing of administration relative to exercise: z = −0.07, p = 0.951). However, these results should be interpreted cautiously because the meta‑analysis included dependent effect sizes and a relatively small number of independent studies, which may limit the reliability of regression‑based tests for publication bias. The certainty of the evidence was downgraded for risk of bias because no study was judged to be at low overall risk of bias and several were rated as high risk, and for indirectness because participants were predominantly male, limiting generalizability to women. Inconsistency was not considered serious due to consistently low or absent heterogeneity across analyses. Imprecision resulted in further downgrading for several outcomes because of small sample sizes and/or confidence intervals crossing or approaching the line of no effect, with very serious imprecision identified for some subgroup analyses. Publication bias was not considered serious because no evidence of publication bias was detected; however, formal assessment was not possible for some subgroup analyses owing to the limited number of studies, and these results should therefore be interpreted cautiously. Overall, the certainty of the evidence ranged from low to very low across outcomes (Supplementary Table 1).

## Discussion

This systematic review and meta-analysis provides updated evidence on the ergogenic potential of caffeine gum on physical performance. Caffeine gum produced a statistically significant but trivial improvement in overall physical performance. Training status significantly moderated this effect, with a small but significant ergogenic benefit observed in trained individuals, whereas no meaningful improvement was evident in recreationally active participants. Across exercise outcomes, caffeine gum elicited the greatest benefit for aerobic endurance, with trivial but significant benefits in anaerobic and jump performance, while muscular strength and endurance showed no significant changes. Although a significant benefit was evident following a 10-min chewing duration, and among individuals with low or moderate habitual caffeine intake, neither chewing duration nor habitual caffeine intake significantly moderated the overall ergogenic response. In addition, higher caffeine doses were associated with greater performance improvements, while the timing of administration relative to exercise had no significant influence on performance.

Beyond providing an updated synthesis of the literature, this meta-analysis extends previous evidence by examining several factors not addressed in earlier reviews. Whereas the previous meta-analysis [30] suggested that caffeine gum was primarily effective when consumed within 15 min before exercise, the present findings indicate that the timing of administration did not significantly moderate the ergogenic response, although evidence beyond the 15-min window remains limited. Furthermore, this is the first meta-analysis to comprehensively evaluate habitual caffeine intake and chewing duration as potential moderators, while also providing a broader assessment across multiple exercise modalities, training status, caffeine dosage, and timing of administration relative to exercise.

The overall standardized mean differences (SMD) observed with caffeine gum were comparable to those reported for caffeine capsules [2]. Consistent with this finding, a study reported that caffeine gum, capsules, and tablets all produced similar improvements in 5-km running performance compared with placebo, with no significant differences between delivery forms despite a slightly larger effect for tablets [72]. Likewise, Ding et al. [54] found that caffeinated chewing gum (4 mg/kg) elicited improvements in maximal strength and muscular power during the bench press and back squat exercises that were comparable to those achieved with caffeine capsules in resistance-trained men. This similarity was expected, as both forms produce equivalent plasma caffeine concentrations despite differing absorption rates [18]. Our findings indicate that caffeine gum significantly enhances physical performance, consistent with the meta-analysis by Barreto et al. [30], which identified caffeine gum as an effective ergogenic strategy for both aerobic and anaerobic exercise. Supporting this, several studies [23, 39] show that consuming caffeine gum 5–15 min before exercise can enhance aerobic endurance [72], cycling time-trial performance [19, 61], repeated sprint ability [19], muscular power (e.g., jumping) [63], maximal strength [38], and team-sport-specific performance [63]. Previous pharmacokinetic studies suggest that the rapid ergogenic effects of caffeine gum may be explained by partial buccal absorption, allowing caffeine to enter the circulation within approximately 5–10 min, together with subsequent gastrointestinal absorption of swallowed caffeine [18]. In addition to systemic mechanisms, oral chemosensory pathways may contribute to the ergogenic effects of caffeine gum. Caffeine can activate bitter taste receptors (TAS2Rs) in the oral cavity, which have been proposed as a potential mechanism underlying the effects of orally administered caffeine, although direct evidence supporting their role in caffeine gum remains limited [73]. Once absorbed, caffeine is thought to enhance exercise performance primarily through adenosine receptor antagonism, increasing arousal, reducing perceived exertion, and enhancing central motor drive [17].

Trained individuals showed a significant ergogenic benefit from caffeine gum supplementation, whereas no meaningful effect was observed in recreationally active participants. This aligns with Barreto et al. [30], who observed clear enhancements in trained but not untrained individuals. One explanation is that trained individuals may be more sensitive to caffeine’s effects on the adenosine system [3]. Indeed, adenosine receptor density (the primary target of caffeine) has been suggested to be higher in trained than untrained individuals [74], potentially increasing sensitivity to caffeine’s antagonism and thereby enhancing central effects such as reduced perception of effort and improved motor drive. The broader literature, however, demonstrates inconsistent ergogenic based on training status. For example, beta-alanine tends to produce smaller effects in highly trained individuals [75], whereas sodium bicarbonate shows diminished effects in untrained participants [76]. Whether caffeine exhibits comparable training status-specific effects remains unclear [77]. This interpretation is limited by the small number of outcomes in the recreationally active group (40 from trained individuals versus 6 from recreationally active participants) and the low total sample size of recreationally active participants (n = 59), which likely reduced statistical power and precision. Beyond caffeine gum administration, the broader caffeine literature suggests that training status is not a consistent moderator of resistance-type outcomes [78]. Accordingly, the apparent trained–untrained differences in our dataset should be interpreted cautiously, as they may reflect sampling and design features rather than systematic physiological moderation by training status.

Current evidence indicates that caffeine gum produces significant improvements in aerobic endurance, anaerobic performance and vertical jump ability, but no meaningful effects on muscular strength or endurance. Consistent with our findings, Barreto et al. [30] reported improvements in exercise performance, particularly for endurance and power-related outcomes. However, our findings diverge for muscular strength, as their meta-analysis identified a positive effect of caffeine gum on strength performance, whereas ours did not. Broader meta-analytic evidence suggests that caffeine exerts greater ergogenic effects in endurance than strength-based tasks [77]. This pattern was also reflected in our findings, where the effect for aerobic endurance outcomes was small, whereas effects for jumping and anaerobic measures were trivial, and muscular strength and endurance outcomes did not show a significant improvement. However, these findings should be interpreted cautiously, as the sensitivity analysis indicated that excluding a single study revealed a small positive effect for muscular strength and endurance, suggesting that the ergogenic benefit may become evident when methodological variability is reduced. Furthermore, while grouping exercise domains facilitated the primary analysis, this approach and the subsequent meta-regression present inherent methodological limitations. Specifically, caffeine's ergogenic effects on strength and power are known to depend on the muscle group involved and contraction mode (e.g., large versus small muscle mass, or eccentric versus concentric actions). Consequently, aggregating these distinct exercise outcomes into broad categories may obscure important physiological differences and potentially mask exercise-specific responses to caffeine. Additional discrepancies among studies may also be attributed to differences in delivery method, timing of ingestion, or participant characteristics [16]. Previous studies have proposed that improvements in endurance performance may be mediated through both central and peripheral mechanisms, whereas improvements in power-related tasks may involve effects on muscle contractile processes. Enhanced sympathetic nervous system activation and increased central motor drive have also been suggested as possible contributors to anaerobic performance [79].

Moderator analysis showed that for the 10 min chewing duration the effect was small but significant, whereas for the 5 min a non-significant impact was revealed. Barreto et al. [30] did not examine chewing duration as a moderator, which makes direct comparison challenging. Nevertheless, individual studies report improvements in physical performance with both shorter [29, 38] and longer chewing [25, 80] periods. To date, no investigation has directly compared these durations; therefore, the findings should be interpreted with caution. As the subgroup estimates were derived from different studies with varying participant characteristics, exercise protocols, and caffeine doses, the apparent differences between chewing durations cannot be attributed solely to chewing time. Nevertheless, the significant effect observed following 10 min of chewing may reflect increased caffeine release and buccal absorption, resulting in greater plasma availability and enhanced adenosine receptor antagonism [17, 31]. In addition, prolonged chewing may increase the exposure of oral taste receptors to caffeine, potentially enhancing the contribution of oral chemosensory pathways, including bitter taste receptor activation, to the ergogenic response [73]. Together, these mechanisms provide a plausible explanation for the greater ergogenic response associated with prolonged chewing on physical performance.

The ergogenic effects of caffeine gum appeared to not depend on habitual caffeine intake. However, individuals with high habitual consumption showed no clear performance benefits, possibly due to tolerance, whereas those with low and moderate intake demonstrated significant improvements. This was reported that chronic caffeine intake may induce tolerance through physiological adaptations, such as the upregulation of adenosine receptors, potentially requiring higher acute doses to elicit similar effects [3]. For example, one study [81] reported that 28 days of caffeine consumption abolishes the performance benefit of 3 mg/kg caffeine. Although caffeine habituation has been proposed to attenuate the ergogenic effects of acute caffeine ingestion, the current evidence is insufficient to draw firm conclusions in habitual high consumers, as the only available evidence was derived from a subgroup analysis comprising just 14 participants, making any conclusions preliminary [3]. In addition, these conclusions must be interpreted with considerable caution due to substantial methodological heterogeneity in the assessment of habitual caffeine intake. Across the current literature, habitual intake was evaluated using a wide variety of tools, including questionnaire-based methods, dietary records, survey-based assessments, and website- or database-based estimation tools. This lack of standardization limits confidence in the observed habituation effects and highlights the need for future research to employ longitudinal, controlled exposure designs.

Caffeine gum doses ranged from 1.35 to 6.4 mg/kg across the included studies. Meta-regression suggested a potential positive association between caffeine dose and effect size, with higher doses generally producing larger ergogenic effects. However, this dose–response relationship was sensitive to analytical assumptions, being non-significant when a within-study correlation of r = 0.2 was assumed (β = 0.083, p = 0.063), but significant at r = 0.8 (β = 0.162, p = 0.008). Leave-one-out sensitivity analyses further indicated that statistical significance was lost when study [22] or study [37] was removed. Our findings are partly consistent with Barreto et al. [30], who reported no significant ergogenic effect at doses <3 mg/kg body mass but a clear benefit at doses ≥3 mg/kg. However, evidence from individual studies remains inconsistent. For example, no improvement in special Judo fitness performance was observed with either 2.7 or 5.4 mg/kg of caffeine gum [60]. Similarly, although 200 mg caffeine gum increased quadriceps strength compared with 100 mg, no dose-dependent effects were found for isometric handgrip or hamstring strength, ball-kicking speed, or 15-s countermovement jump performance [22]. These inconsistencies may reflect differences in participant characteristics, habitual caffeine intake, exercise modality, and study methodology. Taken together, the available evidence suggests a possible dose-dependent ergogenic effect of caffeine gum across the examined dose range (1.35–6.4 mg/kg), although additional well-controlled dose–response investigations are required before optimal dosing recommendations can be made.

Our data did not provide evidence that exercise performance was significantly moderated by the timing of caffeine gum administration before exercise. However, this finding should be interpreted cautiously because the available studies were concentrated within a relatively narrow pre-exercise window. This contrasts with Barreto et al. [30], who reported ergogenic effects only when caffeine gum was consumed within 15 min before exercise. In the present meta-analysis, most outcomes involved caffeine gum administration within this 15-min period (k = 35), whereas only a small number of outcomes examined longer pre-exercise intervals (k = 3). Consequently, the limited variability in administration timing restricted our ability to determine whether timing meaningfully influences the ergogenic response to caffeine gum. Nevertheless, the absence of a significant moderating effect is biologically plausible given the pharmacokinetic characteristics of caffeine gum. Unlike traditional caffeine formulations, caffeine gum exhibits a biphasic plasma concentration profile, with an initial peak occurring within 5–10 min through buccal absorption, followed by a second, more sustained peak approximately 45–105 min post-ingestion due to gastrointestinal absorption of caffeine swallowed with saliva [18]. This dual-peak pattern may maintain effective caffeine availability across a relatively broad pre-exercise period, potentially explaining the lack of a clear timing effect observed in the present analysis. Nonetheless, these findings should not be interpreted as evidence that timing is unimportant. Consistent with a previous meta-analysis [30], evidence examining performance effects during the secondary plasma caffeine peak remains scarce, and the ergogenic significance of this later absorption phase warrants further investigation.

Although most studies were judged to have some concerns regarding risk of bias, with several rated as high risk, several methodological issues warrant consideration. First, all included studies used crossover designs, making washout adequacy, carryover effects, and period effects particularly relevant. Washout periods ranged from 3 to 14 days, with most studies using at least 7 days between experimental conditions. Given the relatively short half-life of caffeine, these intervals were likely sufficient to minimize residual pharmacological effects. However, none of the included studies formally assessed or reported carryover effects or period effects, meaning their potential influence cannot be excluded. Second, successful blinding remains a challenge in caffeine supplementation research because participants may identify the intervention through physiological sensations or expectancy effects. Although placebo gums were often matched for taste, texture, and appearance, several trials did not provide adequately matched placebos, and few formally assessed blinding success. As expectancy effects can influence exercise performance, incomplete blinding may have contributed to the observed ergogenic effects. Therefore, future caffeine-gum trials should incorporate formal assessments of blinding effectiveness and report crossover-specific methodological considerations, including carryover and period effects, to strengthen confidence in the evidence base.

### Practical applications

**Fig. 5:**
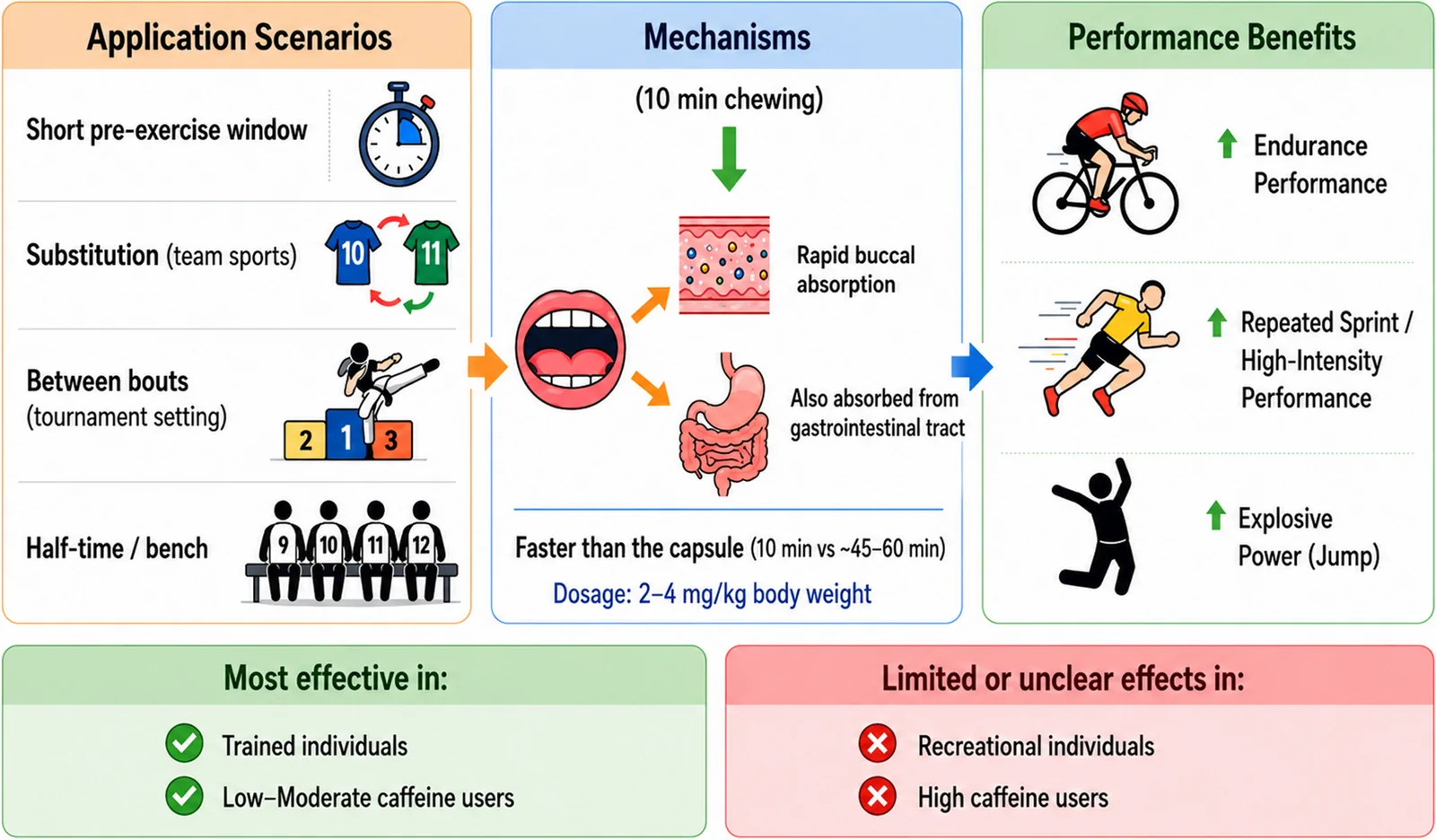
Schematic overview of the applications of caffeine gum in sports, its mechanisms, and associated performance benefits

Caffeine gum may represent a practical strategy for athletes requiring rapid caffeine delivery, particularly when traditional capsules or beverages are impractical (e.g., during half-time, following substitutions, or before early-morning training). Its rapid buccal absorption and high bioavailability may also make it advantageous for longer-duration events by helping to sustain plasma caffeine levels [18]. However, the overall improvement in exercise performance observed in the present meta-analysis was statistically significant but trivial in magnitude (SMD = 0.195). Therefore, although such small effects may be meaningful in elite sport where marginal gains can influence competitive outcomes, the practical benefit is likely to depend on the exercise task, athlete characteristics, and testing context. Figure 5 represents a schematic overview of caffeine gum as a fast-acting strategy in sport and physical performance.

### Limitations

Although this study provides new insights into the effects of caffeine gum on physical performance, several limitations should be acknowledged. First, heterogeneity in training status, exercise type, chewing duration, caffeine habituation, caffeine dosage, and timing of administration relative to exercise across studies may have contributed to variability in effect sizes. Second, most participants were male (359 of 390), which limits the generalizability of the findings, particularly because sex-related differences in caffeine metabolism and pharmacokinetics may influence the ergogenic response. Future studies should recruit sufficient numbers of both male and female participants to directly examine potential sex-specific responses. Third, only English-language studies were included, and grey literature, trial registries, preprints, and theses were not searched, introducing potential for publication and language bias. Fourth, although overall methodological quality was acceptable, concerns were identified regarding the randomization process, blinding procedures, and preregistration practices in some studies. Fifth, the small number of independent studies may compromise the stability and reliability of regression-based publication bias analyses. Sixth, grouping exercise domains simplified the analysis but may have obscured muscle- and contraction-specific responses to caffeine. Additionally, meta-regression across diverse modalities introduced substantial heterogeneity, meaning these findings should be considered exploratory. Finally, converting absolute caffeine doses to relative doses (mg/kg) using mean body mass improved comparability across studies but did not account for individual anthropometric variability, potentially introducing minor imprecision in estimated caffeine exposure.

## Conclusion

This systematic review and meta-analysis demonstrate that caffeine gum produces trivial but statistically significant improvements in physical performance. The rapid buccal absorption of caffeine from chewing gum offers a practical and well-tolerated ergogenic strategy, particularly when traditional caffeine intake is impractical or undesirable. Although the overall magnitude of effect is trivial, these improvements may still be meaningful in competitive settings where marginal gains can influence performance outcomes.

## Supplementary Information

Supplementary Material 1.

## Data Availability

All data relevant to the study are included in the article.

## Abbreviations

SMD: Standardized mean difference
CI: Confidence interval
BM: Body mass
RCTs: Randomized controlled trials
1RM: One‑Repetition Maximum
CYP1A2: Cytochrome P450 1A2 Gene
GRADE: Grading of Recommendations Assessment, Development, and Evaluation
PICO: Population, Intervention, Comparison, Outcome
REML: Restricted Maximum Likelihood
SD: Standard Deviation
PRISMA: Preferred Reporting Items for Systematic Reviews and Meta-Analyses
PROSPERO: International Prospective Register of Systematic Reviews
I^2^: I‑squared (statistical measure of heterogeneity)
Q: Cochran’s Q statistic (test for heterogeneity)
QM: Test statistic for moderators in meta‑analysis (Cochran’s Q for moderators)
QE: Residual heterogeneity statistic (Cochran’s Q for residual heterogeneity)
β: Regression Coefficient (beta coefficient in meta‑regression)
b: Intercept estimate (limit estimate in Egger’s regression)
