# Supplementary material for: Fast-Acting Caffeine Strategy: A Systematic Review and Meta-Analysis of the Ergogenic Effects of Caffeine Chewing Gum on Physical Performance
Source: Sports Med Open. 2026 Sep 9;12:133. doi: 10.1186/s40798-026-01104-y (PMC13558504; doi:10.1186/s40798-026-01104-y)
Supplement: Supplementary file 1 [file 40798_2026_1104_MOESM1_ESM.docx]

**Supplementary table 1: certainty of evidence assessment of studies included in the systematic review.**

| Outcome | Risk of bias | Inconsistency | Indirectness | Imprecision | Publication bias | Final certainty |
| --- | --- | --- | --- | --- | --- | --- |
| Overall exercise performance | Serious. Downgraded because no study was judged as low risk overall, most studies had some concerns, and several were judged as high risk. | Not serious. No downgrade because heterogeneity was low (I² = 4%) and effect estimates were consistent. | Serious. Downgraded because participants were predominantly male, limiting generalisability to women. | Not serious. No downgrade because the pooled estimate was precise and the 95% CI did not cross the line of no effect. | Not serious. Egger’s regression test showed no evidence of funnel plot asymmetry or small-study effects. | Low |
| Recreationally active participants | Serious. Downgraded because no study was judged as low risk overall and several studies were at high risk of bias. | Not serious. No downgrade because heterogeneity was low (I² = 26%). | Serious. Downgraded because participants were predominantly male, limiting generalisability to women. | Very serious. Downgraded two levels because the sample size was small (n = 59) and the 95% CI crossed the line of no effect, indicating substantial uncertainty. | Not serious. Publication bias could not be formally assessed because of the limited number of studies; however, no evidence of publication bias was identified in the overall analysis. | Very low |
| Trained participants | Serious. Downgraded because no study was judged as low risk overall and several studies were at high risk of bias. | Not serious. No downgrade because heterogeneity was absent (I² = 0%). | Serious. Downgraded because participants were predominantly male, limiting generalisability to women. | Not serious. No downgrade because the 95% CI was relatively narrow, did not cross the line of no effect, and the pooled sample size (n = 304) was comparatively large. | Not serious. No evidence of publication bias was detected. | Low |
| High habitual caffeine intake | Serious. Downgraded because no study was judged as low risk overall and several studies were at high risk of bias. | Not serious. No downgrade because heterogeneity was absent (I² = 0%). | Serious. Downgraded because participants were predominantly male, limiting generalisability to women. | Very serious. Downgraded two levels because only two studies/effect sizes (n = 14) were available and the 95% CI crossed the line of no effect. | Not serious. Publication bias could not be formally assessed because of the limited number of studies; however, no evidence of publication bias was identified in the overall analysis. | Very low |
| Low habitual caffeine intake | Serious. Downgraded because no study was judged as low risk overall and several studies were at high risk of bias. | Not serious. No downgrade because heterogeneity was low (I² = 12%). | Serious. Downgraded because participants were predominantly male, limiting generalisability to women. | Serious. Downgraded because the pooled sample size was relatively small (n = 153) and the lower confidence limit was close to the line of no effect. | Not serious. No evidence of publication bias was detected. | Very low |
| Moderate habitual caffeine intake | Serious. Downgraded because no study was judged as low risk overall and several studies were at high risk of bias. | Not serious. No downgrade because heterogeneity was low (I² = 10%). | Serious. Downgraded because participants were predominantly male, limiting generalisability to women. | Serious. Downgraded because the pooled sample size was relatively small (n = 130) and the lower confidence limit was close to the line of no effect. | Not serious. No evidence of publication bias was detected. | Very low |
| Aerobic endurance | Serious. Downgraded because no study was judged as low risk overall and several studies were at high risk of bias. | Not serious. No downgrade because heterogeneity was absent (I² = 0%). | Serious. Downgraded because participants were predominantly male, limiting generalisability to women. | Serious. Downgraded because the pooled sample size was small (n = 122) and the confidence interval remained relatively wide. | Not serious. No evidence of publication bias was detected. | Very low |
| Anaerobic performance | Serious. Downgraded because no study was judged as low risk overall and several studies were at high risk of bias. | Not serious. No downgrade because heterogeneity was absent (I² = 0%). | Serious. Downgraded because participants were predominantly male, limiting generalisability to women. | Serious. Downgraded because the pooled sample size was modest (n = 156) and the lower confidence interval approached the line of no effect. | Not serious. No evidence of publication bias was detected. | Very low |
| Jump performance | Serious. Downgraded because no study was judged as low risk overall and several studies were at high risk of bias. | Not serious. No downgrade because heterogeneity was absent (I² = 0%). | Serious. Downgraded because participants were predominantly male, limiting generalisability to women. | Serious. Downgraded because the pooled sample size was modest (n = 195) and the lower confidence interval approached the line of no effect. | Not serious. No evidence of publication bias was detected. | Very low |
| Muscular strength and endurance | Serious. Downgraded because no study was judged as low risk overall and several studies were at high risk of bias. | Not serious. No downgrade because heterogeneity was low (I² = 35%). | Serious. Downgraded because participants were predominantly male, limiting generalisability to women. | Very serious. Downgraded two levels because the sample size was small (n = 97) and the confidence interval crossed the line of no effect. | Not serious. Publication bias could not be reliably assessed because of the limited number of studies; however, no evidence of publication bias was identified in the overall analysis. | Very low |
| 5-minute chewing duration | Serious. Downgraded because no study was judged as low risk overall and several studies were at high risk of bias. | Not serious. No downgrade because heterogeneity was low (I² = 7%). | Serious. Downgraded because participants were predominantly male, limiting generalisability to women. | Serious. Downgraded because the pooled sample size was relatively small (n = 110) and the lower confidence interval approached the line of no effect. | Not serious. No evidence of publication bias was detected. | Very low |
| 10-minute chewing duration | Serious. Downgraded because no study was judged as low risk overall and several studies were at high risk of bias. | Not serious. No downgrade because heterogeneity was absent (I² = 0%). | Serious. Downgraded because participants were predominantly male, limiting generalisability to women. | Not serious. No downgrade because the confidence interval was reasonably narrow, did not cross the line of no effect, and the effect estimate was relatively precise. | Not serious. No evidence of publication bias was detected. | Low |

**Supplementary table 2. Classification of exercise outcomes according to the predominant energy system or neuromuscular mechanism.**

| Exercise outcome | Category | Justification |
| --- | --- | --- |
| 5-km running | Aerobic endurance | Continuous endurance task predominantly supported by oxidative metabolism. |
| Yo-Yo IR1 | Aerobic endurance | Intermittent endurance test with predominant aerobic contribution. |
| Bruce test | Aerobic endurance | Incremental treadmill protocol assessing aerobic capacity. |
| Treadmill run at 15.4 ± 0.7 km·h⁻¹ | Aerobic endurance | Sustained running task primarily dependent on oxidative metabolism. |
| Cycling to volitional exhaustion | Aerobic endurance | Continuous endurance exercise relying predominantly on aerobic metabolism. |
| 30-km cycling time trial | Aerobic endurance | Prolonged endurance performance primarily supported by oxidative metabolism. |
| Yo-Yo IR2 | Aerobic endurance | Intermittent endurance test with predominant aerobic contribution. |
| RAST | Aerobic endurance | Classified according to the pre-specified outcome grouping used in the meta-analysis. |
| Countermovement jump (CMJ) | Jump performance | Assessment of explosive lower-body jump performance. |
| Sargent jump | Jump performance | Assessment of vertical jump performance. |
| 35-m sprint | Anaerobic performance | Short maximal sprint relying primarily on anaerobic energy pathways. |
| 10 × 40-m sprints | Anaerobic performance | Repeated sprint protocol assessing anaerobic performance. |
| Special Judo Fitness Test | Anaerobic performance | High-intensity intermittent sport-specific performance test. |
| T-Agility Test | Anaerobic performance | High-intensity change-of-direction task relying mainly on anaerobic metabolism. |
| 400-m sprint | Anaerobic performance | Predominantly anaerobic glycolytic exercise. |
| Edgren’s Agility Test | Anaerobic performance | High-intensity agility assessment. |
| 10-m sprint | Anaerobic performance | Maximal acceleration test using ATP-PCr system. |
| 6 × 40-m sprints | Anaerobic performance | Repeated sprint protocol assessing anaerobic performance. |
| Rowing ergometer peak power | Anaerobic performance | Peak power output reflecting short-duration anaerobic performance. |
| 6 × 30-m sprint time | Anaerobic performance | Repeated sprint performance test. |
| Illinois Agility Test | Anaerobic performance | High-intensity agility task. |
| 20-m sprint | Anaerobic performance | Short maximal sprint assessing acceleration. |
| Total number of repetitions | Muscular strength and endurance | Assessment of muscular endurance through repeated contractions to fatigue. |
| 1-RM squat | Muscular strength and endurance | Assessment of maximal dynamic lower-body strength. |
| 1-RM bench press | Muscular strength and endurance | Assessment of maximal dynamic upper-body strength. |
| Handgrip strength | Muscular strength and endurance | Assessment of maximal isometric strength. |
| Romanian deadlift | Muscular strength and endurance | Assessment of maximal posterior-chain strength. |

# **Supplementary table 3. Complete search strategies**

| Database | Complete search strategy | Filters/Limits | Search date | Records retrieved |
| --- | --- | --- | --- | --- |
| PubMed | ("Caffeinated chewing gum" OR "Caffeine gum" OR "Caffeine chewing gum" OR "Caffeinated gum" OR "Caffeine-containing chewing gum") AND ("Exercise performance" OR Performance OR Exercise OR Sport* OR Athlete*) | English; Humans | 11 March 2026 | 71 |
| Web of Science Core Collection | TS=("Caffeinated chewing gum" OR "Caffeine gum" OR "Caffeine chewing gum" OR "Caffeinated gum" OR "Caffeine-containing chewing gum") AND TS=("Exercise performance" OR Performance OR Exercise OR Sport* OR Athlete*) | English; Article | 11 March 2026 | 90 |
| SPORTDiscus (EBSCOhost) | ("Caffeinated chewing gum" OR "Caffeine gum" OR "Caffeine chewing gum" OR "Caffeine-containing chewing gum") AND ("Exercise performance" OR Performance OR Exercise OR Sport* OR Athlete*) | English; Peer-reviewed | 11 March 2026 | 58 |
| Scopus | TITLE-ABS-KEY("Caffeinated chewing gum" OR "Caffeine gum" OR "Caffeine chewing gum" OR "Caffeinated gum" OR "Caffeine-containing chewing gum") AND TITLE-ABS-KEY("Exercise performance" OR Performance OR Exercise OR Sport* OR Athlete*) | English; Article | 11 March 2026 | 76 |

**Supplementary File 4: The following R code was used to perform the analyses reported in the manuscript.**

## Load package

library(metafor)

**Direction-of-effect coding**

Positive effect sizes indicate improved physical performance following caffeine gum ingestion. For outcomes in which lower values represented better performance (e.g., sprint time, running time, cycling time trial, agility tests), the direction of effect was reversed before analysis so that beneficial effects were consistently represented by positive effect sizes. No recoding was applied to outcomes in which higher values indicated better performance (e.g., jump height, distance covered, muscular strength, repetitions, and peak power).

## **Effect size calculation (SMCC)**

data <- escalc( measure="SMCC",
 m1i=gum$`mean-caf`,
 m2i=gum$`mean-pla`,
 sd1i=gum$`sd-caf`,
 sd2i=gum$`sd-pla`,
 ni=gum$`Number of Participants...17`,
 ri=0.5,
 data=gum)

## **Overall multilevel meta-analysis**

odel <- rma.mv( yi=data$yi, V=data$vi,
 random=~1|study_id/effect_id,
 data=data, method="REML")

## **Moderator: Training status**

data$train_status1[data$train_status1 %in% c("NA")] <- NA
result_training <- rma.mv(
 yi=data$yi,V=data$vi, mods=~train_status1,
 random=~1|study_id/effect_id,
 data=data, subset=!is.na(data$train_status1),
 method="REML")

## **Moderator: Exercise outcome**

result_exercise <- rma.mv(
 yi=data$yi,V=data$vi, mods=~Exercise.outcome,
 random=~1|study_id/effect_id,
 data=data, subset=!is.na(data$Exercise.outcome),
 method="REML")

## **Moderator: Habitual caffeine intake**

result_habit <- rma.mv(yi=data$yi,V=data$vi,
 mods=~Caffeine.Habituation.level,
 random=~1|study_id/effect_id,
 data=data, subset=!is.na(data$Caffeine.Habituation.level),
 method="REML")

## **Moderator: Chewing duration**

result_mod <- rma.mv(yi = data$yi,
vi = data$vi, mods = ~ chewing.duration,
random = ~1 | study_id/effect_id,
data = data,subset = !is.na(chewing.duration),
method = "REML")

## **Meta-regression: Dose**

model_dose <- rma.mv(
yi=data$yi, vi=data$vi,
 mods=~Dose.kg,
 random=~1|study_id/effect_id,
 data=data,subset=!is.na(Dose.kg))
summary(model_dose)

## **Meta-regression: Time of Consumption**

data$Time_consumption[data$Time_consumption %in%
c("NA","N/A","na")] <- NA
data$Time_consumption <- as.numeric(data$Time_consumption)

model_time <- rma.mv(
 yi=data$yi,V=data$vi,
 mods=~Time_consumption,
 random=~1|study_id/effect_id,
 data=data,
 subset=!is.na(Time_consumption))
summary(model_time)

## **Sensitivity analysis**

**Sensitivity analysis for assumed within-subject correlation**
data_r02 <- escalc(
measure="SMCC",
m1i=gum$`mean-caf`,
m2i=gum$`mean-pla`,
sd1i=gum$`sd-caf`,
sd2i=gum$`sd-pla`,
ni=gum$`Number of Participants...17`,
ri=0.2,
data=gum)

data_r08 <- escalc(
measure="SMCC",
m1i=gum$`mean-caf`,
m2i=gum$`mean-pla`,
sd1i=gum$`sd-caf`,
sd2i=gum$`sd-pla`,
ni=gum$`Number of Participants...17`,
ri=0.8,
data=gum)

**Leave-one-study-out sensitivity analysis**

studies <- unique(data$study_id)
for(i in studies){
 m <- rma.mv(yi,vi,
 random=~1|study_id/effect_id,
 data=data[data$study_id!=i,])}

## **Publication bias**

**Overall funnel plot**
overall_model <- rma(yi,vi,
data = data2,
method = "REML")

funnel(overall_model)

 **Overall Egger's regression test**
regtest(overall_model)

 **Egger's regression tests for categorical subgroup analyses**

 **Training status**
data2$train_status1 <- as.factor(data2$train_status1)

for(g in levels(data2$train_status1)){

cat("\nTraining status:", g, "\n")

sub <- subset(data2, train_status1 == g)

m <- try(rma(yi, vi, data = sub, method = "REML"), silent = TRUE)

if(!inherits(m, "try-error")){

e <- try(regtest(m), silent = TRUE)

if(!inherits(e, "try-error")){

cat("z =", round(e$zval,3),
"| p =", signif(e$pval,3), "\n")

} else {cat("Egger's test could not be computed.\n")}}}


 **Exercise outcome**
data2$Exercise.outcome <- as.factor(data2$Exercise.outcome)

for(g in levels(data2$Exercise.outcome)){

cat("\nExercise outcome:", g, "\n")

sub <- subset(data2, Exercise.outcome == g)

m <- try(rma(yi, vi, data = sub, method = "REML"), silent = TRUE)

if(!inherits(m, "try-error")){

e <- try(regtest(m), silent = TRUE)

if(!inherits(e, "try-error")){

cat("z =", round(e$zval,3),
"| p =", signif(e$pval,3), "\n")

} else {cat("Egger's test could not be computed.\n")}}}


 **Habitual caffeine intake**
data2$Caffeine.Habituation.level <-
as.factor(data2$Caffeine.Habituation.level)

for(g in levels(data2$Caffeine.Habituation.level)){

cat("\nHabitual caffeine intake:", g, "\n")

sub <- subset(data2,
Caffeine.Habituation.level == g)

m <- try(rma(yi, vi, data = sub, method = "REML"),
silent = TRUE)

if(!inherits(m, "try-error")){

e <- try(regtest(m), silent = TRUE)

if(!inherits(e, "try-error")){

cat("z =", round(e$zval,3),
"| p =", signif(e$pval,3), "\n")

} else {

cat("Egger's test could not be computed.\n")}}}


 **Chewing duration**
data2$chewing.duration <- as.factor(data2$chewing.duration)

for(g in levels(data2$chewing.duration)){

cat("\nChewing duration:", g, "\n")

sub <- subset(data2, chewing.duration == g)

m <- try(rma(yi, vi, data = sub, method = "REML"),
silent = TRUE)

if(!inherits(m, "try-error")){

e <- try(regtest(m), silent = TRUE)

if(!inherits(e, "try-error")){

cat("z =", round(e$zval,3),
"| p =", signif(e$pval,3), "\n")

} else {

cat("Egger's test could not be computed.\n")}}}

**Egger's regression tests for meta-regression models**


 **Caffeine dose**
dose_model <- rma(yi,vi,
mods = ~ Dose.kg,
data = data2,
method = "REML")

regtest(dose_model)


 **Time before exercise**
time_model <- rma(yi,vi,
mods = ~ Time_consumption,
data = data2,
method = "REML")

regtest(time_model)

**Supplementary table 5: Effect Size and Variance Estimates**

|  | Effect Size (yi) | Variance (vi) |
| --- | --- | --- |
| 1 | 0.00000000 | 0.07142857 |
| 2 | 0.30383190 | 0.07472549 |
| 3 | 0.18819649 | 0.07269350 |
| 4 | 0.06477990 | 0.07157844 |
| 5 | 0.17147755 | 0.05637235 |
| 6 | 0.51605393 | 0.06295310 |
| 7 | 0.85365186 | 0.11369673 |
| 8 | 0.09390755 | 0.05580052 |
| 9 | 0.02937736 | 0.12505394 |
| 10 | 0.05839384 | 0.12521312 |
| 11 | 0.09076659 | 0.12551491 |
| 12 | 0.06853245 | 0.05011742 |
| 13 | 0.54534248 | 0.12763325 |
| 14 | 0.12283330 | 0.11194933 |
| 15 | -0.37999553 | 0.07658559 |
| 16 | -0.03803502 | 0.07148024 |
| 17 | 0.31509591 | 0.06997618 |
| 18 | 0.40766780 | 0.07220643 |
| 19 | 0.41255739 | 0.07234012 |
| 20 | 0.26081901 | 0.03829679 |
| 21 | 0.39728289 | 0.07192779 |
| 22 | -0.02702414 | 0.07145465 |
| 23 | 0.21576243 | 0.07309119 |
| 24 | 0.19152929 | 0.05359693 |
| 25 | 0.24596456 | 0.05422365 |
| 26 | 0.48006413 | 0.06195727 |
| 27 | -0.01268219 | 0.08334003 |
| 28 | -0.07421887 | 0.08356285 |
| 29 | 0.01645753 | 0.08334462 |
| 30 | -0.05227680 | 0.07152617 |
| 31 | 0.25494782 | 0.05434206 |
| 32 | 0.40845673 | 0.05702202 |
| 33 | 0.31532275 | 0.06174790 |
| 34 | 0.29839262 | 0.06144230 |
| 35 | 0.52074117 | 0.06679916 |
| 36 | 0.20388802 | 0.06004619 |
| 37 | -0.30462496 | 0.10463982 |
| 38 | 0.27655112 | 0.10382403 |
| 39 | 0.21577947 | 0.10232804 |
| 40 | 0.46276380 | 0.06919220 |
| 41 | 0.33935574 | 0.06609882 |
| 42 | 0.00000000 | 0.06250000 |
| 43 | 0.34819059 | 0.06628865 |
| 44 | 0.24686818 | 0.06440450 |
| 45 | 0.51241593 | 0.07070532 |
| 46 | 0.07646706 | 0.06268273 |
| 47 | -0.48829271 | 0.05890605 |

**Supplementary table 6: Data extracted for study**

| Author | Caffeine Habituation (Tools) | Training status | Caffeine Habituation | Exercise Modalities | Exercise Protocol | Population | Gender | Chewing duration | Time consumption | Dose | Number of Participants | Mean-Caf | Sd-Caf | Mean-Pla | Sd-Pla |
| --- | --- | --- | --- | --- | --- | --- | --- | --- | --- | --- | --- | --- | --- | --- | --- |
| Lynn et.al 2024 | NA | Recreational Active | NA | Aerobic endurance | 5 km Running | Runner | Male | 5 | 30 | 3 | 14 | 1338 | 17.7 | 1338 | 17.7 |
| Tsai et al. 2024 | NA | Trained | NA | Jump | CMJ | Runner | Male | 10 | 10 | 3 | 14 | 47.2 | 4.4 | 45.9 | 3.5 |
| Tsai et al. 2024 | NA | Trained | NA | Anaerobic Performance | 35 - m sprint | Runner | Male | 10 | 10 | 3 | 14 | 6.1 | 0.5 | 6 | 0.5 |
| Tsai et al. 2024 | NA | Trained | NA | Aerobic endurance | Yo-Yo IR1 | Ice Hockey | NA | 10 | 10 | 3 | 14 | 1050 | 300 | 1030 | 280 |
| Farmani et al. 2024 | Questionnaire | Trained | Moderate | Jump | Sargent | TableTennis | Male | 10 | 10 | 3.75 | 18 | 43 | 5.32 | 42 | 5.79 |
| Farmani et al. 2024 | Questionnaire | Trained | Moderate | Aerobic endurance | Bruce Test | TableTennis | Male | 10 | 10 | 3.5 | 18 | 12.263 | 1.304 | 11.58 | 1.22 |
| Dittrich et al. 2021 | Questionnaire | Trained | Moderate | Aerobic endurance | Treadmill Run at 15.4 ± 0.7 km/h | Runner | Male | 5 | NA | 4.2 | 12 | 40.6 | 8.53 | 33.23 | 7.41 |
| Evans et al. 2017 | Questionnaire | Recreational Active | Moderate | Anaerobic Performance | 10x40 m sprints | Team Sport | Male | 10 | 5 | 2.5 | 18 | 87.8 | 3.1 | 87.5 | 3 |
| Ryan et al. 2012 (a) | NA | Recreational Active | Low | Aerobic endurance | Cycling to Volitional Exhaustion | Cyclist | Male | 5 | 35 | 2.35 | 8 | 33.5 | 9.4 | 33.2 | 8.7 |
| Ryan et al. 2012 (b) | NA | Recreational Active | Low | Aerobic endurance | Cycling to Volitional Exhaustion | Cyclist | Male | 5 | 5 | 2.35 | 8 | 33.8 | 9.5 | 33.2 | 8.7 |
| Ryan et al. 2012 (c) | NA | Recreational Active | Low | Aerobic endurance | Cycling to Volitional Exhaustion | Cyclist | Male | 5 | 0 | 2.35 | 8 | 34.5 | 14.6 | 33.2 | 8.7 |
| Paton et al. 2014 | Questionnaire | Trained | Moderate | Aerobic endurance | 30-km cycling time trial | Cyclist | Male/Female | 5 | NA | 3 | 20 | 1100.33 | 128.33 | 1091.33 | 123.66 |
| Filip-Stachnik et al.2021 (a) | Questionnaire | Trained | Moderate | Anaerobic Performance | Special Judo Fitness Test | Judo | Male | 5 | 10 | 6.4 | 9 | 62.22 | 4.32 | 59.66 | 4.15 |
| Filip-Stachnik et al.2021 (b) | Questionnaire | Trained | Moderate | Anaerobic Performance | Special Judo Fitness Test | Judo | Male | 5 | 10 | 6.4 | 9 | 60.22 | 4.08 | 59.66 | 4.15 |
| Yildirim UC et al. 2023 (a) | NA | Trained | High | Jump | CMJ | Soccer | Male | NA | 10 | 1.35 | 14 | 30.2 | 6.31 | 32.43 | 3.95 |
| Yildirim UC et al. 2023 (b) | NA | Trained | High | Jump | CMJ | Soccer | Male | NA | 10 | 2.7 | 14 | 32.23 | 5.55 | 32.43 | 3.95 |
| Liu et al. 2024 (a) | Dietary record | Trained | Low | Anaerobic Performance | T-Agility Test | Basketball | Male | 10 | 10 | 3 | 15 | 10.4 | 0.6 | 10.2 | 0.6 |
| Liu et al. 2024 (b) | Dietary record | Trained | Low | Aerobic endurance | RAST Test | Basketball | Male | 10 | 10 | 3 | 15 | 1354.86 | 44.2 | 1326.7 | 75 |
| Liu et al. 2024 | Dietary record | Trained | Low | Jump | CMJ | Basketball | Male | 10 | 10 | 3 | 15 | 50 | 4 | 48 | 5 |
| Tallis et al 2024 | Survey | NA | Moderate | Jump | CMJ | Rugby | Male | 10 | 10 | 3 | 27 | 33.3 | 6.7 | 31.5 | 6.7 |
| Teimouri-Korani et al. 2025 | Website | Trained | Low | Jump | Sargent | Resistance Training | Male | 10 | 60 | 3.75 | 15 | 41.8 | 5.9 | 39.4 | 5.5 |
| Vargas-Molina et al. 2026 | NA | Trained | NA | Muscle strength and Endurance | Total Number of Repetitions | NA | Male | NA | NA | 3 | 14 | 556 | 147 | 560 | 130 |
| Vargas-Molina et al. 2026 | NA | Trained | NA | jump | CMJ | NA | Male | NA | NA | 6 | 14 | 40.9 | 7.31 | 39.3 | 6.59 |
| Yi-Jie Shiu 2024 | NA | Trained | NA | jump | CMJ | Sprinter | Male | 10 | 15 | 3 | 19 | 55 | 5 | 54 | 5 |
| Yi-Jie Shiu 2024 | NA | Trained | NA | Anaerobic Performance | 400 -m sprint | Sprinter | Male | 10 | 15 | 3 | 19 | 54.68 | 2.88 | 53.87 | 3.37 |
| Pirmohammadi et al. 2023 | NA | Trained | NA | Anaerobic Performance | Edgren’s Agility Test | TableTennis | FEMALE | 10 | 10 | 3.75 | 18 | 24.38 | 2.19 | 23.22 | 2.41 |
| Kaszuba et al. 2023 (a) | Questionnaire | Trained | Moderate | Anaerobic Performance | T-Agility Test | Volleyball | Male/Female | 5 | 15 | 3.2 | 12 | 9.44 | 0.69 | 9.45 | 0.77 |
| Kaszuba et al. 2023 (b) | Questionnaire | Trained | Moderate | Anaerobic Performance | 10 m sprint | Volleyball | Male/Female | 5 | 15 | 3.2 | 12 | 1.68 | 0.13 | 1.69 | 0.12 |
| Kaszuba et al. 2023 | Questionnaire | Trained | Moderate | Jump | CMJ | Volleyball | Male/Female | 5 | 15 | 3.2 | 12 | 51.2 | 11.2 | 51 | 11.4 |
| Russell et al. 2017 | NA | Trained | Moderate | Anaerobic Performance | 6 × 40 m sprints w | Rugby | Male | 5 | 10 | 4 | 14 | 6.06 | 0.18 | 6.07 | 0.18 |
| Veiner et al. 2019 | NA | Trained | Low | jump | CMJ | Resistance Training | Male | 10 | NA | 3 | 19 | 36.4 | 6.2 | 34.8 | 5.8 |
| Veiner et al. 2019 | NA | Trained | Low | Anaerobic Performance | Rowing Ergometer Peak Power | Resistance Training | Male | 10 | 0 | 3.61 | 19 | 667.5 | 78.5 | 635.9 | 68.7 |
| Ranchordas et al. 2019 (a) | NA | Trained | NA | Anaerobic Performance | 6*30m Sprint Time | Rugby | Male | 5 | 0 | 2.35 | 17 | 4.6 | 0.28 | 4.5 | 0.32 |
| Ranchordas et al. 2019 (b) | NA | Trained | NA | Anaerobic Performance | Illinois Agility Test | Rugby | Male | 5 | 0 | 2.35 | 17 | 16.22 | 1.08 | 15.88 | 1.09 |
| Ranchordas et al. 2019 | NA | Trained | NA | Aerobic endurance | Yo-Yo IR2 | Rugby | Male | 5 | 0 | 2.35 | 17 | 426 | 105 | 372 | 91 |
| Ranchordas et al. 2019 | NA | Trained | NA | Jump | CMJ | Rugby | Male | 5 | 10 | 3 | 17 | 43.7 | 7.6 | 42.2 | 6.2 |
| Ranchordas et al. 2018 | NA | Trained | Low | Anaerobic Performance | 20 Meter Sprint | Soccer | Male | 5 | 5 | 2.66 | 10 | 3.1 | 0.3 | 3.2 | 0.3 |
| Ranchordas et al. 2018 | NA | Trained | Low | Jump | CMJ | Soccer | Male | 5 | 10 | 3 | 10 | 47.1 | 3.4 | 46.1 | 3.2 |
| Ranchordas et al. 2018 | NA | Trained | Low | Aerobic endurance | Yo-Yo IR1 | Soccer | Male | 5 | 5 | 2.66 | 10 | 1754 | 156 | 1719 | 139 |
| Li Ding et al. 2025 (a) | Questionnaire | Trained | Low | Muscle strength and Endurance | 1 RM Strength Squat | Resistance Training | Male | 5 | 5 | 4 | 16 | 172.3 | 20.2 | 161.9 | 22.3 |
| Li Ding et al. 2025 (b) | Questionnaire | Trained | Low | Muscle strength and Endurance | 1 RM Strength Bench Press | Resistance Training | Male | 5 | 5 | 4 | 16 | 105.3 | 14.5 | 100.3 | 13.4 |
| Li Ding et al. 2025 (a) | Questionnaire | Trained | Low | Muscle strength and Endurance | 1 RM Bench Press | Resistance Training | Male | 5 | 5 | 3 | 16 | 94 | 12 | 94 | 12 |
| Li Ding et al. 2025 (b) | Questionnaire | Trained | Low | Muscle strength and Endurance | 1 RM Squat | Resistance Training | Male | 5 | 5 | 3 | 16 | 160 | 18 | 153 | 20 |
| Li Ding et al. 2025 (a) | Questionnaire | Trained | Low | Muscle strength and Endurance | 1 RM Bench Press | NA | Male | NA | 5 | 4 | 16 | 96 | 12 | 93 | 11 |
| Li Ding et al. 2025 (b) | Questionnaire | Trained | Low | Muscle strength and Endurance | 1 RM Squat | Resistance Training | Male | NA | 5 | 4 | 16 | 168 | 19 | 158 | 18 |
| Tzeng et al.2026 | Dietary records | Trained | Low | Muscle strength and Endurance | Hand grip | wrestlers | Male | 10 | 15 | 3 | 16 | 102.1 | 12.1 | 101 | 14.8 |
| Chen et al.2023 | NA | Recreational Active | Low | Muscle strength and Endurance | Romanian deadlift | Resistance Training | NA | 5 | 10 | 2.5 | 19 | 487 | 158.5 | 585.4 | 214.9 |
